# Heat Stress-Derived Plasma Extracellular Vesicles Protect Hepatocytes in Chickens by Suppressing MYD88/NF-κB/MAPK Signaling

**DOI:** 10.3390/cells15090836

**Published:** 2026-05-02

**Authors:** Zi Mei, Haobo Zhou, Chaoyang Gao, Hao Du, Kunyuan Liu, Zheya Sheng, Yanzhang Gong

**Affiliations:** Key Laboratory of Agricultural Animal Genetics, Breeding and Reproduction, Ministry of Education, College of Animal Science and Technology and College of Veterinary Medicine, Huazhong Agricultural University, Wuhan 430070, China

**Keywords:** heat stress, extracellular vesicles, liver, inflammatory response, poultry

## Abstract

**Highlights:**

**What are the main findings?**

- Heat stress increased the release of plasma-derived extracellular vesicles (EVs) in chickens, and these EVs preferentially accumulated in the liver and were efficiently internalized by primary hepatocytes.
- Heat stress-derived plasma EVs alleviated hepatocyte injury under thermal stress by restoring proliferation, reducing apoptosis, and attenuating inflammatory signaling, which was associated with suppression of the MYD88/NF-κB/MAPK pathway.

**What are the implications of the main findings?**

- Circulating EVs may function as adaptive intercellular messengers during heat stress rather than merely passive by-products of tissue injury.
- Heat stress-derived plasma EVs may represent an endogenous mediator of hepatoprotection and provide a basis for developing EV-based strategies to mitigate heat stress-induced liver injury in poultry.

**Abstract:**

Heat stress is a major systemic challenge in poultry, but the role of circulating extracellular vesicles (EVs) in liver-directed adaptation remains unclear. This study investigated whether plasma-derived EVs from heat-stressed chickens (HS_EV) mediate hepatoprotective responses under thermal stress. EVs were isolated from the plasma of control and heat-stressed chickens and characterized by morphology, size distribution, and marker expression. Their biodistribution in vivo and uptake by primary hepatocytes in vitro were also evaluated. Hepatocyte injury was induced by heat exposure, and the effects of HS_EV on proliferation, apoptosis, inflammatory cytokine production, transcriptomic reprogramming, and MYD88/NF-κB/MAPK signaling were assessed. Heat stress induced systemic injury in chickens, increased the release of plasma-derived EVs, and promoted their preferential accumulation in the liver. Whole-transcriptome analysis further showed that HS_EV enhanced glutathione metabolism and related metabolic pathways while suppressing apoptosis- and inflammation-related signaling. In primary hepatocytes, HS_EV, but not control EVs, restored proliferative capacity, reduced apoptosis, suppressed the expression and secretion of IL-1β, IL-6, and TNF-α under heat stress, and was associated with attenuation of the MYD88/NF-κB/MAPK axis. These findings suggest that circulating EVs participate in adaptive intercellular communication during heat stress and identify HS_EV as a potential endogenous mediator of hepatoprotection in chickens.

## 1. Introduction

Heat stress is an increasingly important challenge to poultry production in the context of global warming and intensive farming [1,2,3,4,5]. Because chickens are covered with feathers, lack sweat glands, and have limited heat-dissipation capacity, they are especially susceptible to elevated ambient temperatures [6,7]. Excessive heat exposure disrupts physiological homeostasis and triggers oxidative stress, metabolic disturbance, endocrine imbalance, immune dysfunction, and inflammatory activation, ultimately impairing performance and causing multi-organ injury [1,2,8,9]. Thus, heat stress is now widely regarded as a systemic pathological challenge rather than a simple environmental burden.

Among the organs affected by heat stress, the liver is particularly vulnerable because of its central roles in metabolism, detoxification, and immunoregulation [10,11,12,13,14]. Previous studies have shown that heat stress can induce hepatic steatosis, oxidative injury, inflammation, and apoptosis, indicating that the liver is a major target organ during thermal challenge [12,15,16]. However, although heat stress-induced liver injury has been increasingly recognized, the mechanisms by which systemic stress signals are transmitted to hepatocytes and reshape hepatic responses remain poorly understood.

Extracellular vesicles (EVs) have emerged as important mediators of intercellular communication under both physiological and pathological conditions [17,18,19]. These nanosized vesicles carry diverse bioactive cargos, including proteins, lipids, mRNAs, miRNAs, and other non-coding RNAs, and can alter recipient-cell behavior after uptake [20,21,22,23]. Importantly, EV release, cargo composition, and biological activity can be dynamically altered by environmental stress, suggesting that they may serve as circulating messengers in systemic adaptation [24,25]. Consistent with this idea, systemically delivered extracellular vesicles are often enriched in the liver, and hepatocytes are capable of actively internalizing extracellular vesicles and responding to their cargos. In addition, extracellular vesicles have been implicated in hepatic inflammation, apoptosis, regeneration, and metabolic remodeling in multiple injury settings [26]. In the present study, we use the term EVs because the specific subcellular origin and biogenesis pathway of the isolated vesicles were not directly demonstrated.

Accumulating evidence indicates that heat stress triggers systemic inflammatory responses and metabolic disturbances in the liver [12,15]. Activation of stress-related signaling pathways, including the Toll-like receptor (TLR), nuclear factor kappa B (NF-κB), and mitogen-activated protein kinase (MAPK) pathways, contributes to hepatocellular dysfunction and apoptosis [27,28,29,30]. In particular, myeloid differentiation primary response 88 (MYD88), a key adaptor of TLR signaling, plays a central role in amplifying inflammatory cascades [31,32,33,34]. However, the upstream regulatory mechanisms that modulate hepatic inflammatory signaling under heat stress conditions remain incompletely understood. Despite growing interest in stress-associated EVs, it is still unclear whether circulating EVs contribute to hepatic adaptation or injury during thermal challenge in poultry.

Based on these considerations, we hypothesized that heat stress promotes the release of circulating plasma EVs in chickens and that these EVs participate in liver-directed adaptive responses to thermal injury. Therefore, this study aimed to isolate and characterize plasma-derived EVs from control and heat-stressed chickens, determine their biodistribution and hepatocyte uptake, and evaluate their effects on hepatocyte proliferation, apoptosis, inflammatory signaling, and cytokine production under heat stress conditions. By integrating transcriptomic and non-coding RNA profiling with molecular validation, we further sought to explore the mechanisms associated with EV-mediated hepatoprotection, with particular focus on the MYD88/NF-κB/MAPK axis.

## 2. Materials and Methods

### 2.1. Animals and Experimental Design

A total of 80 28-week-old Xinhua No. 2 laying hens were obtained from Hubei Xinhua Ecological Animal Husbandry Development Co., Ltd. (Xiaogan, China). Before heat exposure, all birds were acclimated for 2 weeks under standard conditions (23 ± 1 °C, 60–70% relative humidity, 15 h light/9 h dark).

The heat stress trial was conducted in a climate-controlled chamber at the experimental poultry farm of Huazhong Agricultural University, where temperature (±0.5 °C) and humidity (~60%) were monitored throughout the experiment. Based on our previous work, acute heat stress was induced by exposing hens to 36 °C for 6 h. The birds were randomly assigned to two groups: a control group (23 ± 1 °C, *n* = 40) and a heat stress group (36 °C for 6 h, *n* = 40). Feed and water were available ad libitum during the experiment.

### 2.2. Phenotypic Measurements and Sample Collection

At the end of the 6 h treatment, rectal temperature was recorded in all birds. Blood was collected from the wing vein for physiological and biochemical analyses. Serum was obtained by centrifugation (2500× *g*, 15 min, 4 °C) and stored at −80 °C until use. Blood gas variables were analyzed using i-STAT CG8+/EG8+ cartridges (Abbott, Princeton, NJ, USA), and serum biochemical parameters were measured with an automated chemistry analyzer (BX-4000, Sysmex, Kobe, Japan).

For tissue sampling, four hens from each group were randomly selected and euthanized. Heart, liver, spleen, lung, and kidney tissues were collected immediately. Part of each tissue was fixed in 4% paraformaldehyde for histology, whereas the remaining portion was snap-frozen in liquid nitrogen and stored at −80 °C for further analysis.

### 2.3. Histological Analysis

For histological examination, heart, liver, spleen, lung, and kidney tissues from four chickens per group (*n* = 4) were fixed in 4% paraformaldehyde for 24 h at room temperature. Samples were then dehydrated in graded ethanol, cleared in xylene, and embedded in paraffin. Paraffin blocks were sectioned at 6 μm using a microtome (Leica RM2235, Leica Biosystems Nussloch GmbH, Nussloch, Germany) and stained with hematoxylin and eosin (H&E). Sections were observed under an Olympus BX53 light microscope (Olympus Corporation, Tokyo, Japan), and representative images were processed with ImageJ software (version 1.53; National Institutes of Health, Bethesda, MD, USA).

### 2.4. Isolation of Plasma-Derived Extracellular Vesicles

Plasma-derived extracellular vesicles (EVs) were isolated by differential ultracentrifugation. Plasma was first centrifuged at 1500× *g* for 12 min at 4 °C to remove residual blood cells. The recovered supernatant was diluted 1:1 with ice-cold PBS and sequentially centrifuged at 2000× *g* for 30 min and 12,000× *g* for 45 min at 4 °C to eliminate cell debris and larger particles. The supernatant was then passed through a 0.22 μm filter, diluted with PBS, and ultracentrifuged at 100,000× *g* for 75 min at 4 °C. The pellet was resuspended in pre-chilled PBS, filtered again through a 0.22 μm membrane, and subjected to a second ultracentrifugation at 100,000× *g* for 2 h at 4 °C. The final EV pellet was resuspended in 20–50 μL PBS and stored at −80 °C until further use.

### 2.5. Transmission Electron Microscopy (TEM)

EV morphology was evaluated by transmission electron microscopy (TEM). EVs were resuspended in 2% paraformaldehyde, placed onto Formvar-carbon-coated copper grids, and allowed to adsorb for 10 min. The grids were subsequently washed with PBS, fixed with 1% glutaraldehyde for 5 min, rinsed with ddH_2_O/PBS for 2 min, and negatively stained with uranyl acetate for approximately 10 min on ice. After air-drying for 5–10 min, the samples were examined using a Hitachi HT7800 transmission electron microscope (Hitachi High-Tech Corporation, Tokyo, Japan) operated at 120 kV.

### 2.6. Nanoparticle Tracking Analysis (NTA)

EV size distribution and concentration were determined by nanoparticle tracking analysis (NTA) using a ZetaVIEW instrument (Particle Metrix GmbH, Inning am Ammersee, Germany). Samples were diluted 1:200 in PBS and measured at 28.82 °C and pH 7.0 with a 520 nm laser in scatter mode. Data acquisition and analysis were performed with ZetaView software (version 8.05.14 SP7) at ×10 magnification, with a frame rate of 30 fps, gain 24, and shutter setting 70. The analysis settings were: minimum brightness = 20, minimum area = 5, maximum area = 1000, and minimum trace length = 15. The average particle count per frame was 243.8, and the total number of traces was 1660. Particle size was expressed as the median diameter (D50/X50), and concentration was calculated automatically by the software.

### 2.7. Western Blot Analysis

Protein expression in primary hepatocytes and plasma-derived EVs was assessed by Western blotting. Samples were lysed in RIPA buffer (Epizyme, Shanghai, China; PC101) containing 1% PMSF (Epizyme, Shanghai, China; GRF101) and 1% phosphatase inhibitor (Beyotime, Shanghai, China), and the lysates were centrifuged at 12,000× *g* for 15 min at 4 °C. Protein concentration was measured using a BCA protein assay kit (Epizyme, Shanghai, China; ZJ102). Equal amounts of protein were separated by 10–15% SDS-PAGE, transferred to PVDF membranes (Millipore, Billerica, MA, USA), and blocked with QuickBlock™ blocking buffer (Beyotime, Guangzhou, China; P0240) for 15 min. Membranes were incubated overnight at 4 °C with primary antibodies against CD44, ALIX, TSG101, CD63, MYD88, p-p65, p65, p-ERK, ERK1/2, p-JNK, JNK, p-p38, p38, and β-actin, followed by incubation with HRP-conjugated goat anti-rabbit IgG (AS014, ABclonal, Wuhan, China; 1:5000) or HRP-conjugated goat anti-mouse IgG (AS013, ABclonal, Wuhan, China; 1:5000) for 2 h at room temperature. Chemiluminescent signals were developed using the Omni-ECL™ Femto Light kit (Epizyme, Shanghai, China; SQ201) and quantified with Quantity One software (version 4.6.2; Bio-Rad Laboratories, Hercules, CA, USA).

### 2.8. In Vivo Tracking of EVs

To determine the in vivo distribution of plasma-derived EVs, PKH67-labeled EVs were injected into the wing vein at 1 mg/kg body weight. After injection, the puncture site was gently pressed until bleeding or leakage was no longer visible, and the birds were returned to their cages. Twenty-four hours later, chickens were euthanized and the heart, liver, spleen, lung, and kidney were collected for fluorescence examination. Fresh tissues were embedded in OCT compound, cryosectioned, rinsed with PBS, counterstained with DAPI, and mounted with anti-fade medium. Fluorescence signals were then visualized and recorded under a fluorescence microscope.

### 2.9. Isolation and Identification of Primary Hepatocytes

Primary hepatocytes were isolated from freshly collected chicken liver by collagenase IV digestion. Liver tissue was aseptically excised, washed with ice-cold PBS containing 1% penicillin–streptomycin, and minced into small fragments before enzymatic digestion at 37 °C. The digested suspension was filtered through a 70 μm cell strainer and collected by low-speed centrifugation. After washing, the cells were resuspended in DMEM/F12 medium supplemented with 10% fetal bovine serum and 1% penicillin–streptomycin, and maintained at 37 °C in a humidified incubator with 5% CO_2_. Hepatocyte identity was confirmed by cell morphology together with PAS staining and CK18 immunofluorescence.

### 2.10. In Vitro Uptake of EVs

For the cellular uptake assay, primary hepatocytes were seeded in 24-well plates and exposed to PKH67-labeled plasma-derived EVs at a final concentration of 20 μg/mL for 24 h. After incubation, cells were washed with ice-cold PBS, fixed with 4% paraformaldehyde for 10–15 min at room temperature, and rinsed again with PBS. Nuclei were counterstained with DAPI, and the cells were mounted with anti-fade medium. Fluorescence microscopy was used to assess intracellular uptake of labeled EVs.

### 2.11. Establishment of a Heat Stress Model in Primary Hepatocytes

To establish the in vitro heat stress model, primary chicken hepatocytes were cultured for 24 h and then exposed to 43 °C for 0, 2, 4, 6, or 8 h. Control cells were maintained at 37 °C. Based on cell viability, LDH release, and stress-related protein expression, 43 °C for 4 h was selected as the standard heat stress condition for subsequent experiments.

### 2.12. LDH Assay

LDH activity in culture supernatants was measured using a commercial kit (Nanjing Jiancheng Bioengineering Institute, Nanjing, China). After treatment, supernatants were collected and clarified by centrifugation (4000 rpm, 5 min). LDH detection was then performed according to the kit protocol, and absorbance was recorded at 440 nm using a microplate reader.

### 2.13. ELISA for Inflammatory Cytokines

The concentrations of IL-1β, IL-6, and TNF-α in culture supernatants were determined by ELISA using commercial kits (Nanjing Jiancheng Bioengineering Institute, Nanjing, China). Supernatants were harvested after treatment, centrifuged to remove cell debris, and used for cytokine measurement. Absorbance was measured with a microplate reader, and cytokine concentrations were calculated from the corresponding standard curves.

### 2.14. RNA Extraction and Quality Control

Total RNA was isolated from primary hepatocytes using TRIzol reagent (Invitrogen, Carlsbad, CA, USA). RNA concentration and purity were assessed with a NanoDrop spectrophotometer (Thermo Fisher Scientific, Waltham, MA, USA), and RNA integrity was evaluated using an Agilent 2100 Bioanalyzer (Agilent Technologies, Santa Clara, CA, USA). Only samples with RIN ≥ 7.0 were used for library preparation and sequencing.

### 2.15. Whole-Transcriptome Sequencing

Primary hepatocytes treated with control or heat stress-derived plasma EVs were assigned to the Ctrl_EV and HS_EV groups, respectively, with five biological replicates in each group. Total RNA was extracted after treatment, and ribosomal RNA was removed using the Ribo-Zero Gold rRNA Removal Kit (Illumina Inc., San Diego, CA, USA). The resulting rRNA-depleted RNA was fragmented and used to construct libraries with the TruSeq Stranded Total RNA Library Prep Kit (Illumina Inc., San Diego, CA, USA). Library quality and concentration were evaluated using an Agilent 2100 Bioanalyzer (Agilent Technologies, Santa Clara, CA, USA) and a Qubit fluorometer (Thermo Fisher Scientific, Waltham, MA, USA), respectively, and qualified libraries were subjected to Illumina sequencing (Illumina Inc., San Diego, CA, USA).

### 2.16. Small RNA Sequencing

For small RNA profiling, the same Ctrl_EV- and HS_EV-treated hepatocyte samples were used, with five biological replicates per group. One microgram of total RNA from each sample was used for library construction with the TruSeq Small RNA Sample Prep Kit (Illumina, Cat. No. RS-200-0012). After adapter ligation, reverse transcription, and PCR amplification, library quality was evaluated using DNA High Sensitivity Chips on an Agilent 2100 Bioanalyzer (Agilent Technologies, Santa Clara, CA, USA). Qualified libraries were sequenced on an Illumina HiSeq X Ten platform.

### 2.17. Identification of Differentially Expressed mRNAs, lncRNAs, circRNAs, and miRNAs

Clean reads from whole-transcriptome sequencing were aligned to the chicken reference genome (*Gallus gallus*, GRCg7b) using HISAT2 (v2.2.1), and transcript assembly and quantification were performed with StringTie (v2.2.3). Differentially expressed mRNAs were identified using the criteria |log2FoldChange| > 1 and adjusted *p* value (padj) < 0.05. For lncRNA identification, transcripts shorter than 200 bp and known protein-coding transcripts were removed, and the remaining transcripts were evaluated for coding potential using CNCI (v2.0), CPC2 (v3.2.0), and Pfam-scan (v1.6). Candidate lncRNAs were then screened for differential expression using the same thresholds as those applied to mRNAs. CircRNAs were identified from unmapped reads using CIRI (v2.0.6) and find_circ (v1.2), and their abundance was normalized as transcripts per million (TPM). For miRNA analysis, clean small RNA reads were aligned to the reference sequence using Bowtie (v1.0.1). The remaining reads were used for miRNA quantification and normalized to TPM. Differentially expressed circRNAs and miRNAs were defined using |log2FoldChange| > 1 and padj < 0.05.

### 2.18. Target Prediction of Differentially Expressed ncRNAs

To explore the regulatory potential of differentially expressed non-coding RNAs, target genes were predicted for lncRNAs, circRNAs, and miRNAs. Protein-coding genes located within 100 kb upstream or downstream of a given lncRNA were considered potential cis-regulatory targets. CircRNA–miRNA interactions were predicted with miRanda (v3.3a) based on putative miRNA binding sites within circRNA sequences. For miRNA–mRNA targeting, predictions were performed using miRanda (v3.3a) and RNAhybrid (v2.0) according to complementary binding between miRNAs and the 3′UTR of mRNAs.

### 2.19. Gene Ontology (GO) and Kyoto Encyclopedia of Genes and Genomes (KEGG) Pathway Enrichment Analyses

GO and KEGG enrichment analyses were carried out using the clusterProfiler package (v4.8.1) in R (v4.5.2). Significant GO terms and KEGG pathways among differentially expressed genes or predicted target genes were identified using the hypergeometric test. A *p* value < 0.05 was considered statistically significant.

### 2.20. Gene Set Enrichment Analysis

Gene set enrichment analysis (GSEA) was performed using the clusterProfiler package (v4.8.1) in R (v4.5.2) to evaluate pathway-level changes across the whole transcriptome. All genes were ranked according to their differential expression between groups, and KEGG pathway gene sets were used as the reference database. The normalized enrichment score (NES), nominal *p* value, and false discovery rate (FDR) were calculated for each pathway. Pathways with nominal *p* < 0.05 and *FDR* < 0.25 were considered significantly enriched.

### 2.21. Construction of ceRNA Regulatory Networks

ceRNA networks were constructed to investigate potential post-transcriptional interactions among differentially expressed lncRNAs, circRNAs, miRNAs, and mRNAs. Candidate lncRNA–miRNA, circRNA–miRNA, and miRNA–mRNA interaction pairs were first obtained by sequence-based prediction. For the lncRNA-associated network, differentially expressed lncRNAs, miRNAs, and mRNAs were integrated according to target prediction results and expression correlations to generate putative lncRNA–miRNA–mRNA triplets. CircRNA-associated ceRNA networks were built using the same strategy based on predicted circRNA–miRNA interactions, miRNA–mRNA targeting relationships, and correlation analysis. Only RNA pairs with a correlation coefficient > 0.85 and *p* < 0.05 were retained, and the final networks were visualized with Cytoscape (v3.10.3).

### 2.22. RNA Reverse Transcription and cDNA Synthesis

For mRNA analysis, cDNA was synthesized from 1 μg of total RNA using the HiScript^®^ III RT SuperMix Kit (Vazyme, Nanjing, China; R323-01) after removal of genomic DNA. Reverse transcription was performed at 55 °C for 15 min and terminated at 85 °C for 15 s.

For miRNA analysis, stem-loop reverse transcription was performed using 1 μg of total RNA after genomic DNA removal. Mature miRNA sequences were obtained from miRBase, and primers for selected miRNAs and gga-5S rRNA were synthesized by Tsingke Biotechnology Co., Ltd. (Wuhan, China). The reaction program was 25 °C for 5 min, 50 °C for 15 min, and 85 °C for 5 min. Primer sequences are provided in Table A1.

### 2.23. Quantitative Real-Time PCR

Gene expression was validated by quantitative real-time PCR (qRT-PCR) using a Bio-Rad CFX-384 system (Bio-Rad Laboratories, Hercules, CA, USA) and 2× SYBR Green Fast qPCR Mix (ABclonal Technology Co., Ltd., Wuhan, China). Each sample was analyzed in triplicate. The amplification program consisted of 95 °C for 3 min, followed by 40 cycles of 95 °C for 10 s and 60 °C for 30 s. Melting curve analysis was performed to confirm amplification specificity. Relative mRNA and miRNA expression levels were calculated using the 2^−ΔΔCt^ method. β-actin was used as the internal reference for mRNA, whereas gga-5S rRNA was used for miRNA normalization. Primer sequences are listed in Table A1 and Table A2.

### 2.24. EV Treatment of Primary Hepatocytes

To examine the effects of plasma-derived EVs on heat stress-induced hepatocyte injury, primary hepatocytes were divided into four groups: Blank, HS, HS + Ctrl_EV, and HS + HS_EV. Cells in the Blank group were maintained under normal culture conditions, whereas the HS group was exposed to heat stress alone. For EV pretreatment, hepatocytes were incubated with Ctrl_EV or HS_EV at a final concentration of 20 μg/mL for 24 h before heat exposure. Cells were then subjected to 43 °C for 4 h, and cells or culture supernatants were collected for CCK-8, EdU, TUNEL, qRT-PCR, Western blot, and ELISA analyses.

### 2.25. MYD88 Plasmid Transfection

The full-length coding sequence of chicken MYD88 was cloned into the pcDNA3.1 expression vector, and the empty vector served as the negative control. Primary chicken hepatocytes were transfected with the MYD88-pcDNA3.1 plasmid or empty vector using Lipofectamine 3000 (Invitrogen, Carlsbad, CA, USA) according to the manufacturer’s instructions. For the rescue experiment, hepatocytes were divided into five groups: Blank, HS, HS + HS_EV, HS + HS_EV + Vector, and HS + HS_EV + MYD88. Cells were pretreated with HS_EV (20 μg/mL, 24 h) and then transfected with the indicated plasmids before heat stress exposure (43 °C for 4 h). Transfection efficiency was verified by qRT-PCR and Western blotting.

### 2.26. PMA Treatment

Phorbol 12-myristate 13-acetate (PMA; MedChemExpress, HY-18739) was used as an NF-κB activator and dissolved in DMSO. For the rescue experiment, primary chicken hepatocytes were divided into five groups: Blank, HS, HS + HS_EV, HS + HS_EV + Vehicle, and HS + HS_EV + PMA. Cells in the HS + HS_EV, HS + HS_EV + Vehicle, and HS + HS_EV + PMA groups were treated with HS_EV (20 μg/mL) for 24 h. PMA (20 ng/mL) was added simultaneously with HS_EV in the HS + HS_EV + PMA group, whereas an equal volume of DMSO was added to the HS + HS_EV + Vehicle group. After treatment, cells were exposed to heat stress at 43 °C for 4 h, and samples were collected for CCK-8, EdU, TUNEL, qRT-PCR, Western blot, and ELISA analyses.

### 2.27. Cell Counting Kit-8 Assay

Cell proliferation was assessed using the Cell Counting Kit-8 (CCK-8; Dojindo, Kumamoto, Japan; CK04). Primary hepatocytes (5 × 10^3^ cells/well) were seeded into 96-well plates and exposed to the indicated treatments. Absorbance at 450 nm was recorded at 24, 48, and 72 h using a microplate reader.

### 2.28. Ethynyldeoxyuridine (EdU) Assay

Cell proliferative activity was further evaluated by EdU incorporation. After treatment, primary hepatocytes were incubated with EdU working solution for 2 h at 37 °C, fixed with 4% paraformaldehyde, permeabilized with 0.5% Triton X-100, and then reacted with the click chemistry solution in the dark. Nuclei were counterstained with DAPI, and fluorescence images were obtained under a fluorescence microscope. The percentage of EdU-positive cells was used as an index of proliferation.

### 2.29. TUNEL Assay

Apoptosis was examined using a TUNEL staining assay. Following the indicated treatments, primary hepatocytes were fixed with 4% paraformaldehyde, permeabilized with 0.1% Triton X-100, and incubated with the TUNEL reaction mixture at 37 °C in the dark. After washing, nuclei were counterstained with DAPI. Fluorescence microscopy was used to visualize TUNEL-positive cells, and the apoptotic rate was calculated accordingly.

### 2.30. Statistical Analysis

All experiments were independently repeated at least three times. Data are presented as mean ± SD. Statistical analyses were performed using GraphPad Prism 8.0. Comparisons between two groups were conducted using Student’s *t*-test, whereas one-way ANOVA followed by Tukey’s multiple-comparisons test was used for comparisons among multiple groups. In the figures, different lowercase letters indicate statistically significant differences among multiple groups, whereas asterisks indicate significant differences in pairwise comparisons (* *p* < 0.05, ** *p* < 0.01, *** *p* < 0.001). A *p* value < 0.05 was considered statistically significant.

## 3. Results

### 3.1. Heat Stress Disrupts Physiological Homeostasis and Triggers Systemic Stress and Inflammatory Responses in Chickens

**Figure 1:**
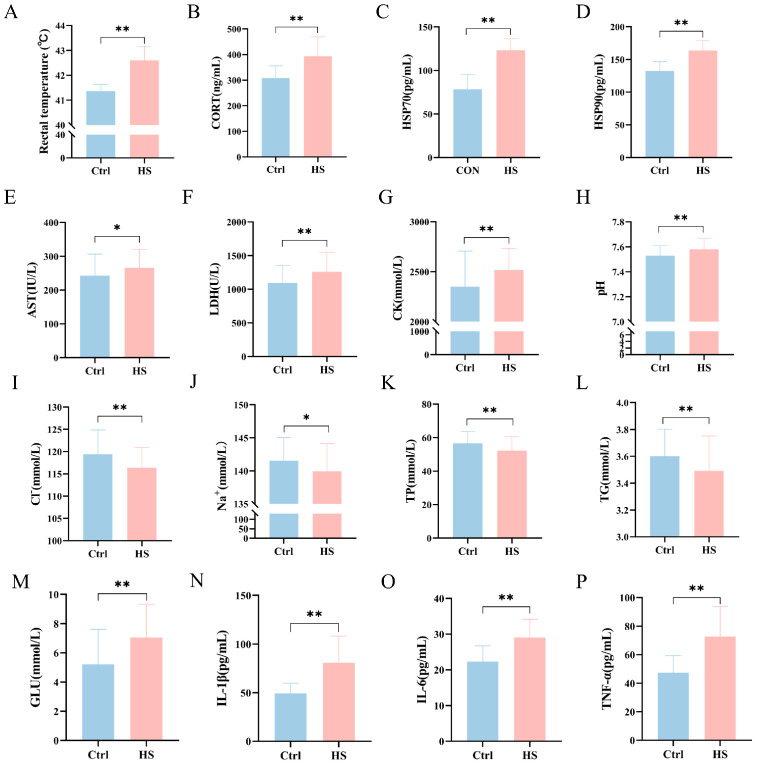
Effects of heat stress on physiological, biochemical, metabolic, and inflammatory parameters in chickens (*n* = 40 per group). (**A**) Rectal temperature; (**B**) CORT; (**C**) HSP70; (**D**) HSP90; (**E**) LDH; (**F**) AST; (**G**) CK; (**H**) pH; (**I**) Cl^−^; (**J**) Na^+^; (**K**) TG; (**L**) TP; (**M**) GLU; (**N**) IL-1β; (**O**) IL-6; and (**P**) TNF-α. Data are presented as mean ± SD. * *p* < 0.05, ** *p* < 0.01.

Compared with the control (Ctrl) group, heat-stressed (HS) chickens exhibited a significantly increased rectal temperature (*p* < 0.05), indicating successful induction of systemic thermal stress (Figure 1A). Blood biochemical analysis further showed that the levels of corticosterone (CORT), heat shock protein 70 (HSP70), heat shock protein 90 (HSP90), lactate dehydrogenase (LDH), aspartate aminotransferase (AST), and creatine kinase (CK) were all significantly elevated (*p* < 0.05), reflecting activation of systemic stress responses and tissue injury under heat stress conditions (Figure 1B–G). In addition, plasma pH was significantly increased, whereas chloride (Cl^−^) and sodium (Na^+^) concentrations were significantly decreased (Figure 1H–J).

Heat stress also markedly altered circulating metabolic and inflammatory indices. Specifically, triglyceride (TG) and total protein (TP) levels were significantly reduced, whereas glucose (GLU) concentration was significantly increased (*p* < 0.05) (Figure 1K–M). Meanwhile, the circulating pro-inflammatory cytokines tumor necrosis factor-α (TNF-α), interleukin-6 (IL-6), and interleukin-1β (IL-1β) were all significantly increased (*p* < 0.05) (Figure 1N–P). Collectively, these results indicate that heat stress disrupted physiological homeostasis and induced systemic stress, metabolic disturbance, and inflammatory activation in chickens.

### 3.2. Heat Stress Is Associated with Multiorgan Histopathological Alterations in Chickens

**Figure 2:**
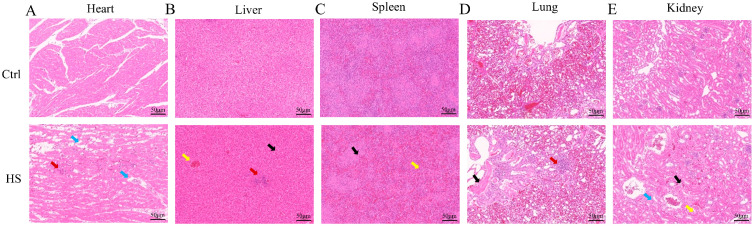
Histopathological changes in the heart, liver, spleen, lung, and kidney of chickens under heat stress. Representative hematoxylin and eosin (H&E)-stained sections of the (**A**) heart, (**B**) liver, (**C**) spleen, (**D**) lung, and (**E**) kidney from the Ctrl and HS groups (*n* = 4 per group, scale bar = 50 μm). Colored arrows indicate representative histopathological alterations in different organs. Red arrows indicate inflammatory cell infiltration or inflammatory foci; blue arrows indicate increased adipocytes or loosening of epithelial cell cytoplasm; yellow arrows indicate congestion or round vacuoles; and black arrows indicate round vacuoles, uneven white pulp distribution, vascular congestion, or extravasated erythrocytes.

Histopathological examination of tissues collected from 4 chickens per group (*n* = 4) showed that heat stress induced mild-to-moderate lesions in multiple organs. In the heart, adipocyte infiltration and occasional focal cardiomyocyte necrosis were observed (Figure 2A). In the liver, hepatic steatosis, cytoplasmic vacuolization, scattered inflammatory foci, and venous congestion were evident, although the hepatic cord architecture remained largely preserved (Figure 2B). The spleen displayed white pulp atrophy and red pulp congestion (Figure 2C), whereas the lung showed mild hemorrhage (Figure 2D). In the kidney, tubular lipid vacuolization and vascular congestion were observed without obvious glomerular injury (Figure 2E). Collectively, these findings provide morphological evidence of systemic organ injury under heat stress, accompanied by metabolic disturbance, vascular congestion, and low-grade inflammation, with the liver showing particularly evident pathological alterations.

### 3.3. Isolation, Characterization, and In Vivo Hepatic Distribution of Heat Stress-Derived Plasma EVs

**Figure 3:**
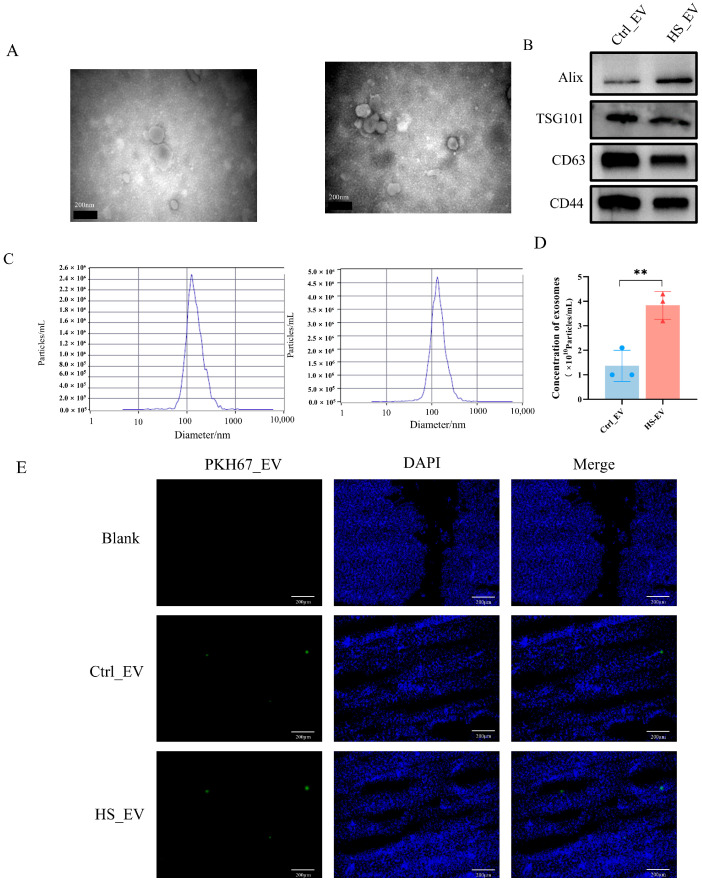
Isolation, characterization, and hepatic distribution of plasma-derived EVs from Ctrl and HS chickens. (**A**) Transmission electron microscopy images of Ctrl_EV and HS_EV (*n* = 4, scale bar = 200 nm). (**B**) Western blot analysis of EV-associated marker proteins (*n* = 3). (**C**) Nanoparticle tracking analysis of EV size distribution (*n* = 3). (**D**) Quantification of EV protein concentration (*n* = 3). (**E**) Representative fluorescence images of liver sections after intravenous administration of PKH67-labeled EVs (*n* = 5, scale bar = 200 μm). Ctrl_EV, plasma-derived EVs from control chickens; HS_EV, plasma-derived EVs from heat-stressed chickens. Data are presented as mean ± SD. ** *p* < 0.01.

Plasma-derived EVs were isolated from Ctrl and HS chickens by differential ultracentrifugation. TEM showed that the isolated vesicles exhibited the typical cup-shaped morphology and bilayer membrane structure of EVs (Figure 3A). Western blot analysis confirmed the presence of the common EV-associated markers ALIX, TSG101, CD63, and CD44 in both groups (Figure 3B), and NTA showed that particle diameters were mainly distributed between 30 and 140 nm (Figure 3C). In addition, BCA quantification showed that the total EV protein content was significantly increased in the plasma of HS chickens compared with the Ctrl group (Figure 3D), indicating enhanced EV release under heat stress conditions.

**Figure A1:**
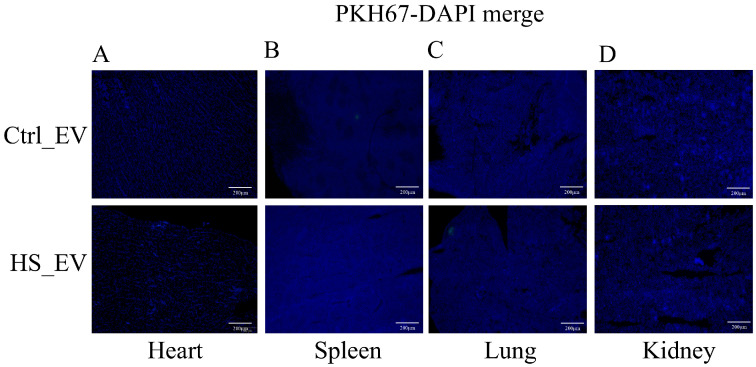
In vivo distribution of PKH67-labeled plasma-derived EVs in non-hepatic tissues of chickens. Representative fluorescence images of tissue sections collected 24 h after intravenous injection of PKH67-labeled plasma-derived EVs. (**A**) Heart, (**B**) spleen, (**C**) lung, and (**D**) kidney.

To examine tissue distribution in vivo, PKH67-labeled plasma-derived EVs were intravenously injected into chickens. At 24 h after injection, fluorescence signals were predominantly detected in the liver, whereas weaker signals were observed in the heart, spleen, lung, and kidney (Figure 3E and Figure A1). These results indicate that circulating plasma-derived EVs preferentially accumulated in the liver in vivo.

### 3.4. Efficient Internalization of Plasma-Derived EVs by Primary Hepatocytes In Vitro

**Figure 4:**
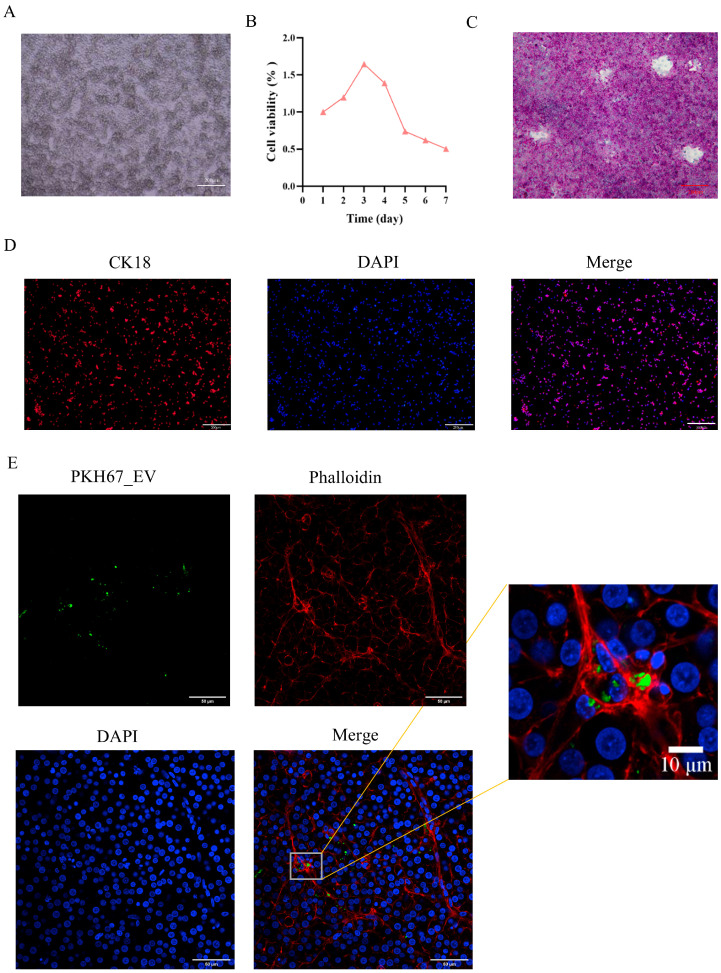
Characterization of primary chicken hepatocytes and uptake of plasma-derived EVs in vitro. (**A**) Bright-field image of primary hepatocytes (*n* = 3, scale bar = 200 μm). (**B**) Cell viability of hepatocytes during culture (*n* = 8). (**C**) PAS staining of primary hepatocytes (*n* = 3, scale bar = 200 μm). (**D**) CK18 immunofluorescence staining of primary hepatocytes (*n* = 3, scale bar = 200 μm). (**E**) Uptake of PKH67-labeled plasma-derived EVs by primary hepatocytes (*n* = 3). Scale bars are indicated in the corresponding panels (50 μm and 10 μm for the enlarged view).

To further verify the cellular uptake of plasma-derived EVs in vitro, primary hepatocytes were first isolated and identified. As shown in Figure 4A, the isolated cells exhibited the typical polygonal morphology of hepatocytes. Cell viability analysis further showed that the cultured hepatocytes maintained good viability during the early culture period (Figure 4B). In addition, PAS staining demonstrated abundant glycogen deposition in the cytoplasm (Figure 4C), and CK18 immunofluorescence staining was strongly positive (Figure 4D), confirming the hepatocytic identity of the isolated cells.

To assess EV uptake, primary hepatocytes were incubated with PKH67-labeled plasma-derived EVs for 24 h. Fluorescence imaging revealed clear intracellular green signals in hepatocytes, which were mainly localized in the cytoplasmic region and delineated by phalloidin-labeled cytoskeletal structures (Figure 4E). The merged and enlarged images further confirmed the internalization of PKH67-labeled EVs by primary hepatocytes. These results indicate that plasma-derived EVs can be efficiently internalized by primary hepatocytes in vitro, thereby supporting subsequent functional analyses under heat stress conditions.

### 3.5. Whole-Transcriptome Sequencing Quality of Hepatocytes Treated with Ctrl_EV and HS_EV

Whole-transcriptome sequencing was performed in primary hepatocytes treated with Ctrl_EV or HS_EV, with five biological replicates per group. For rRNA-depleted RNA sequencing, a total of 137.96 Gb of clean data was obtained (Table 1). All samples showed Q20 and Q30 values above 90%, and the mapping rates to the chicken reference genome exceeded 81%, indicating high sequencing quality and good comparability among samples. For small RNA sequencing, a total of 110.9 million clean reads was generated (Table 2), with GC contents ranging from 53% to 56%, Q20 and Q30 values above 97.6%, and mapping rates exceeding 93% in all samples. Collectively, these results indicate that both the transcriptome and small RNA sequencing datasets were of high quality and suitable for subsequent analyses.

### 3.6. Heat Stress-Derived Plasma EVs Induce Transcriptomic Alterations in Primary Hepatocytes

**Figure 5:**
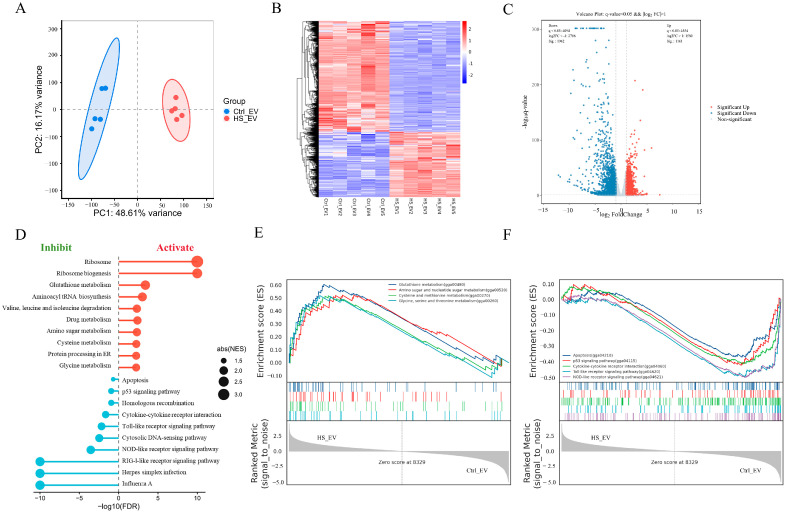
Transcriptomic alterations in primary hepatocytes treated with Ctrl_EV or HS_EV under heat stress (*n* = 5). (**A**) Principal component analysis (PCA) of transcriptomic profiles. (**B**) Heatmap of differentially expressed genes. (**C**) Volcano plot of differentially expressed genes. (**D**) Summary of gene set enrichment analysis (GSEA) results. (**E**) Representative GSEA plot showing a positively enriched pathway in the HS_EV group. (**F**) Representative GSEA plot showing a negatively enriched pathway in the HS_EV group.

To investigate the molecular responses of hepatocytes to heat stress-derived plasma EVs (HS_EV), whole-transcriptome sequencing was performed in primary hepatocytes treated with Ctrl_EV or HS_EV. Principal component analysis (PCA) showed clear separation between the two groups, with PC1 and PC2 explaining 92.41% and 2.81% of the total variance, respectively, indicating marked differences in global transcriptional profiles (Figure 5A). Hierarchical clustering analysis also revealed distinct gene expression patterns between Ctrl_EV- and HS_EV-treated hepatocytes (Figure 5B).

**Figure A2:**
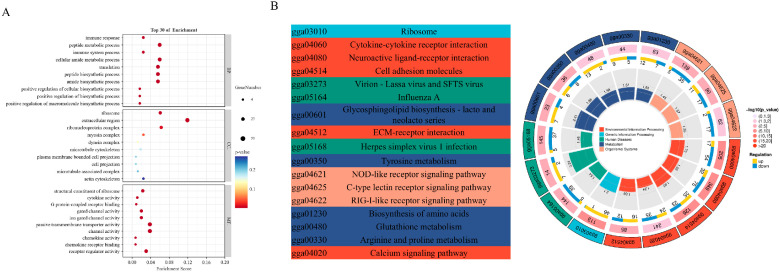
GO and KEGG enrichment analyses of differentially expressed mRNAs in primary hepatocytes treated with Ctrl_EV or HS_EV under heat stress. (**A**) Top 30 significantly enriched GO terms. (**B**) Significantly enriched KEGG pathways.

Using the thresholds of |log2FC| > 1 and FDR < 0.05, a total of 3123 differentially expressed genes (DEGs) were identified in the HS_EV group relative to the Ctrl_EV group, including 1161 upregulated and 1962 downregulated genes (Figure 5C). GO enrichment analysis showed that these DEGs were mainly associated with immune response, peptide metabolic process, cellular amide metabolic process, translation, and peptide biosynthetic process, whereas KEGG analysis highlighted ribosome, cytokine-cytokine receptor interaction, cell adhesion molecules, glutathione metabolism, and several inflammation- and metabolism-related pathways (Figure A2). Gene set enrichment analysis (GSEA) further showed that HS_EV significantly activated glutathione metabolism, amino sugar and nucleotide sugar metabolism, cysteine and methionine metabolism, and glycine, serine and threonine metabolism, while apoptosis, the p53 signaling pathway, cytokine-cytokine receptor interaction, the Toll-like receptor signaling pathway, and the NOD-like receptor signaling pathway were significantly suppressed (Figure 5D–F). Collectively, these findings indicate that HS_EV induced extensive transcriptomic reprogramming in primary hepatocytes, particularly in pathways associated with inflammatory signaling, apoptosis, and metabolic adaptation.

### 3.7. HS_EV Reshapes the Non-Coding RNA Landscape and Associated Functional Pathways in Primary Hepatocytes

**Figure 6:**
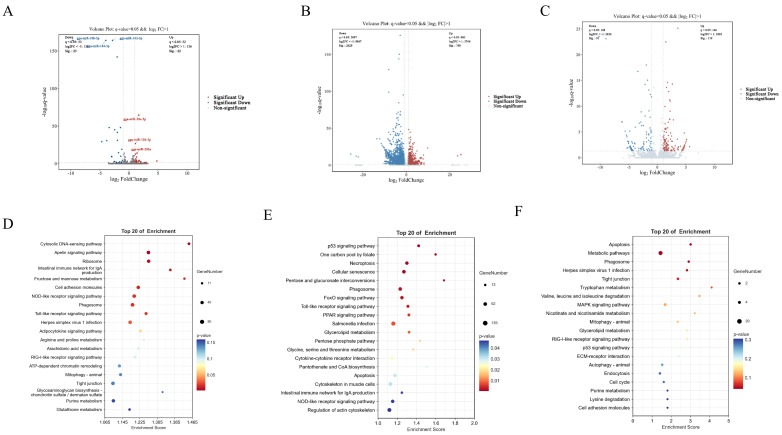
Differential expression and KEGG enrichment analyses of non-coding RNAs in primary hepatocytes treated with Ctrl_EV or HS_EV under heat stress (*n* = 5). (**A**–**C**) Volcano plots of differentially expressed miRNAs, lncRNAs, and circRNAs, respectively. (**D**–**F**) Top 20 KEGG pathways enriched for miRNA target genes, lncRNA-associated target genes, and circRNA host genes, respectively.

**Figure A3:**
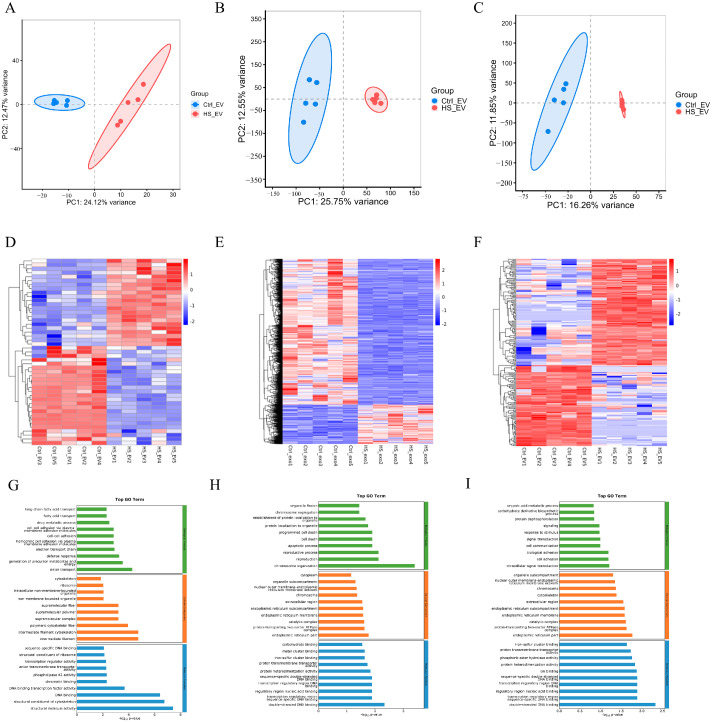
PCA, hierarchical clustering, and GO enrichment analyses of differentially expressed non-coding RNAs in primary hepatocytes treated with Ctrl_EV or HS_EV under heat stress. (**A**–**C**) Principal component analysis (PCA) of miRNAs, lncRNAs, and circRNAs, respectively. (**D**–**F**) Hierarchical clustering heatmaps of differentially expressed miRNAs, lncRNAs, and circRNAs, respectively. (**G**–**I**) Top enriched GO terms of miRNA target genes, lncRNA-associated target genes, and circRNA host genes.

To further characterize the regulatory effects of heat stress-derived plasma EVs (HS_EV) on hepatocytes, the expression profiles of miRNAs, lncRNAs, and circRNAs were analyzed. Principal component analysis and hierarchical clustering showed clear separation between the Ctrl_EV and HS_EV groups at all three non-coding RNA levels, indicating distinct expression patterns induced by HS_EV (Figure A3A–F). Differential expression analysis identified 47 differentially expressed miRNAs (DEMs), including 22 upregulated and 25 downregulated transcripts, 244 differentially expressed lncRNAs (DELs), including 102 upregulated and 142 downregulated transcripts, and 210 differentially expressed circRNAs (DECs), including 119 upregulated and 91 downregulated transcripts (Figure 6A–C). These results indicate that HS_EV induced extensive reprogramming of the non-coding RNA landscape in primary hepatocytes.

To further explore the potential functions of these HS_EV-responsive non-coding RNAs, KEGG enrichment analysis was performed on miRNA target genes, lncRNA-associated target genes, and circRNA host genes. For miRNAs, the enriched pathways mainly included the NOD-like receptor signaling pathway, Toll-like receptor signaling pathway, phagosome, and cell adhesion molecules. For lncRNAs, the corresponding target genes were significantly associated with the p53 signaling pathway, Toll-like receptor signaling pathway, NOD-like receptor signaling pathway, apoptosis, and cytokine-cytokine receptor interaction. For circRNAs, the host genes were significantly enriched in apoptosis, the MAPK signaling pathway, the RIG-I-like receptor signaling pathway, the p53 signaling pathway, phagosome, and metabolic pathways (Figure 6D–F). In addition, GO enrichment results are presented in Figure A3. Integrated analysis further showed that these differentially expressed miRNAs, lncRNAs, and circRNAs converged on pathways associated with inflammation, apoptosis, stress responses, and metabolic regulation (Figure A3G–I). In particular, the Toll-like receptor, NOD-like receptor, RIG-I-like receptor, MAPK, and p53 signaling pathways were repeatedly enriched, suggesting that HS_EV-responsive non-coding RNAs may participate in the coordinated regulation of inflammatory and apoptotic signaling in primary hepatocytes. The enrichment of multiple metabolism-related pathways further indicates a potential role for these non-coding RNAs in hepatocellular metabolic adaptation following HS_EV treatment.

**Figure A4:**
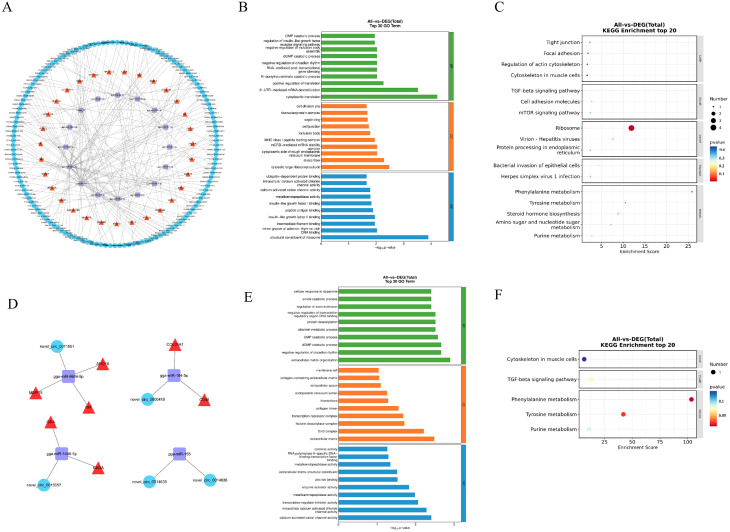
ceRNA network construction and enrichment analyses of differentially expressed lncRNAs and circRNAs. (**A**) lncRNA–miRNA–mRNA network. (**B**,**C**) GO and KEGG enrichment analyses of lncRNA-related target genes. (**D**) circRNA–miRNA–mRNA network. (**E**,**F**) GO and KEGG enrichment analyses of circRNA-related target genes.

**Figure A5:**
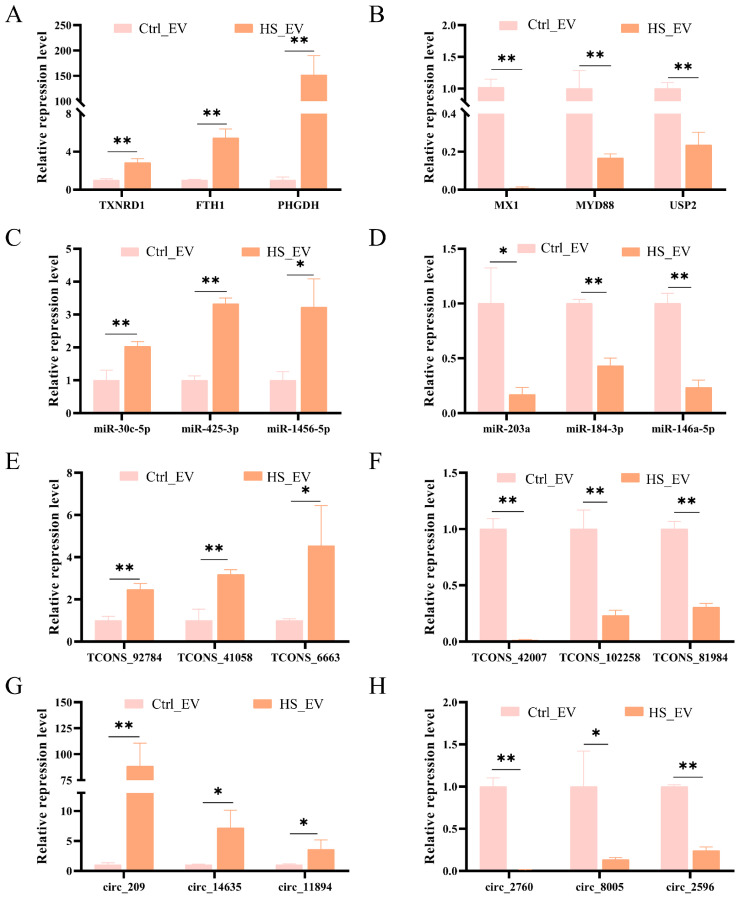
qRT-PCR validation of selected differentially expressed mRNAs, miRNAs, lncRNAs, and circRNAs identified by sequencing (*n* = 4). (**A**,**B**) mRNAs; (**C**,**D**) miRNAs; (**E**,**F**) lncRNAs; (**G**,**H**) circRNAs. (**A**,**C**,**E**,**G**) show upregulated transcripts, whereas (**B**,**D**,**F**,**H**) show downregulated transcripts in the HS_EV group relative to the Ctrl_EV group. Data are presented as mean ± SD. * *p* < 0.05, ** *p* < 0.01.

Moreover, ceRNA network analysis provided complementary evidence that both lncRNA- and circRNA-associated regulatory networks may be involved in HS_EV-responsive hepatocyte reprogramming, with the lncRNA-associated ceRNA network appearing more complex and extensive than the circRNA-associated network (Figure A4). qRT-PCR validation of representative mRNAs and non-coding RNAs further confirmed the sequencing results, with the selected transcripts showing expression changes consistent with the corresponding sequencing data (Figure A5). Collectively, these findings support the reliability and reproducibility of the transcriptomic and small RNA sequencing data.

### 3.8. Establishment of a Heat Stress-Induced Hepatocyte Injury Model In Vitro

**Figure 7:**
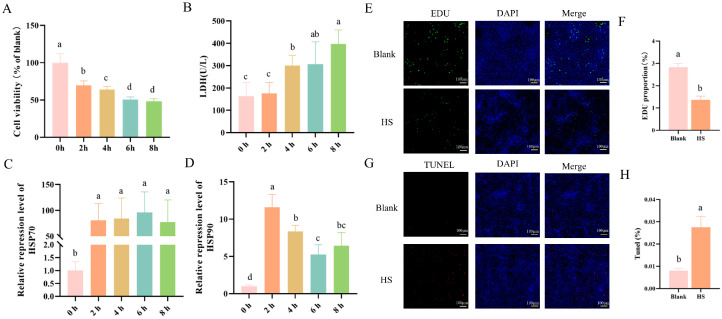
Establishment of the heat stress-induced injury model in primary hepatocytes. (**A**) Cell viability (*n* = 8). (**B**) LDH release (*n* = 8). (**C**,**D**) Relative expression of HSP70 and HSP90 after heat stress for 0, 2, 4, 6, and 8 h (*n* = 4). (**E**,**F**) EdU staining and quantification (*n* = 3, scale bar = 100 μm). (**G**,**H**) TUNEL staining and quantification (*n* = 3, scale bar = 100 μm). Data are presented as mean ± SD. Different lowercase letters indicate significant differences among groups or time points (*p* < 0.05).

To establish a reliable in vitro model for evaluating the functional effects of heat stress-derived plasma EVs (HS_EV), primary hepatocytes were exposed to 43 °C for 0, 2, 4, 6, and 8 h, and cellular injury was assessed by measuring cell viability, lactate dehydrogenase (LDH) release, and the expression of heat shock proteins HSP70 and HSP90. As the duration of heat exposure increased, cell viability progressively declined, with significantly lower viability observed after 2, 4, 6, and 8 h compared with the 0 h group (Figure 7A). In parallel, LDH activity in the culture supernatant gradually increased during heat stress and reached the highest level at 8 h, indicating progressive membrane damage and cellular injury (Figure 7B). The expression levels of HSP70 and HSP90 were both markedly induced by heat stress. HSP70 expression increased rapidly after heat exposure and remained significantly elevated throughout the treatment period (Figure 7C). In contrast, HSP90 expression peaked at 2 h and then gradually declined, although it remained significantly higher than that in the 0 h group (Figure 7D). These findings indicate that heat stress effectively activated the cellular stress response in primary hepatocytes.

Based on these results, 43 °C for 4 h was selected as the heat stress condition for subsequent experiments. Under this condition, EdU staining showed that the proportion of proliferating hepatocytes was significantly reduced compared with that in the Blank group, indicating that heat stress markedly suppressed hepatocyte proliferative activity (Figure 7E,F). In contrast, TUNEL staining revealed a significant increase in the proportion of apoptotic cells in the heat-stressed group relative to the Blank group, indicating that heat stress markedly promoted hepatocyte apoptosis (Figure 7G,H). Collectively, these results indicate that exposure to 43 °C for 4 h established a robust hepatocyte injury model characterized by reduced proliferative activity and enhanced apoptosis.

### 3.9. Heat Stress-Derived Plasma EVs Alleviate Heat Stress-Induced Hepatocyte Injury

**Figure 8:**
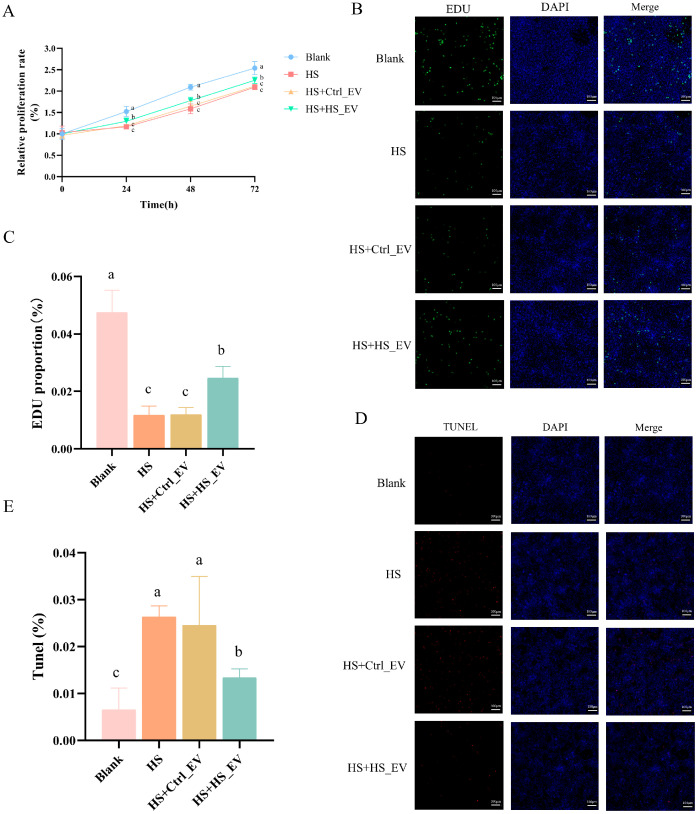
Effects of Ctrl_EV and HS_EV on proliferation and apoptosis of primary hepatocytes under heat stress. (**A**) Relative proliferation rate (*n* = 8). (**B**,**C**) EdU staining and quantification (*n* = 3, scale bar = 100 μm). (**D**,**E**) TUNEL staining and quantification (*n* = 3, scale bar = 100 μm). Data are presented as mean ± SD. Different lowercase letters indicate significant differences among groups (*p* < 0.05).

To further investigate the effects of plasma-derived EVs on heat stress-induced hepatocyte injury, four experimental groups were established: Blank, HS, HS + Ctrl_EV, and HS + HS_EV. CCK-8 assays showed that heat stress significantly inhibited hepatocyte proliferation at 24, 48, and 72 h compared with the Blank group. EV treatment partially alleviated this inhibitory effect, with the HS + HS_EV group exhibiting a more pronounced increase in proliferation rate than the HS and HS + Ctrl_EV groups (Figure 8A).

Consistently, EdU staining showed that heat stress markedly reduced the proportion of proliferating hepatocytes. No obvious improvement was observed in the HS + Ctrl_EV group compared with the HS group, whereas HS + HS_EV treatment significantly increased the proportion of EdU-positive cells, indicating partial restoration of proliferative capacity (Figure 8B,C). TUNEL staining further showed that heat stress markedly increased hepatocyte apoptosis relative to the Blank group. A comparable apoptotic level was observed in the HS + Ctrl_EV group, whereas the HS + HS_EV group exhibited a significantly lower proportion of TUNEL-positive cells than both the HS and HS + Ctrl_EV groups (Figure 8D,E). Collectively, these results indicate that HS_EV alleviated heat stress-induced hepatocyte injury by restoring proliferative activity and reducing apoptosis, whereas Ctrl_EV showed little or no obvious protective effect under the same conditions.

### 3.10. HS_EV Inhibits Heat Stress-Induced Activation of the MYD88/NF-κB/MAPK Signaling Pathway in Primary Hepatocytes

**Figure 9:**
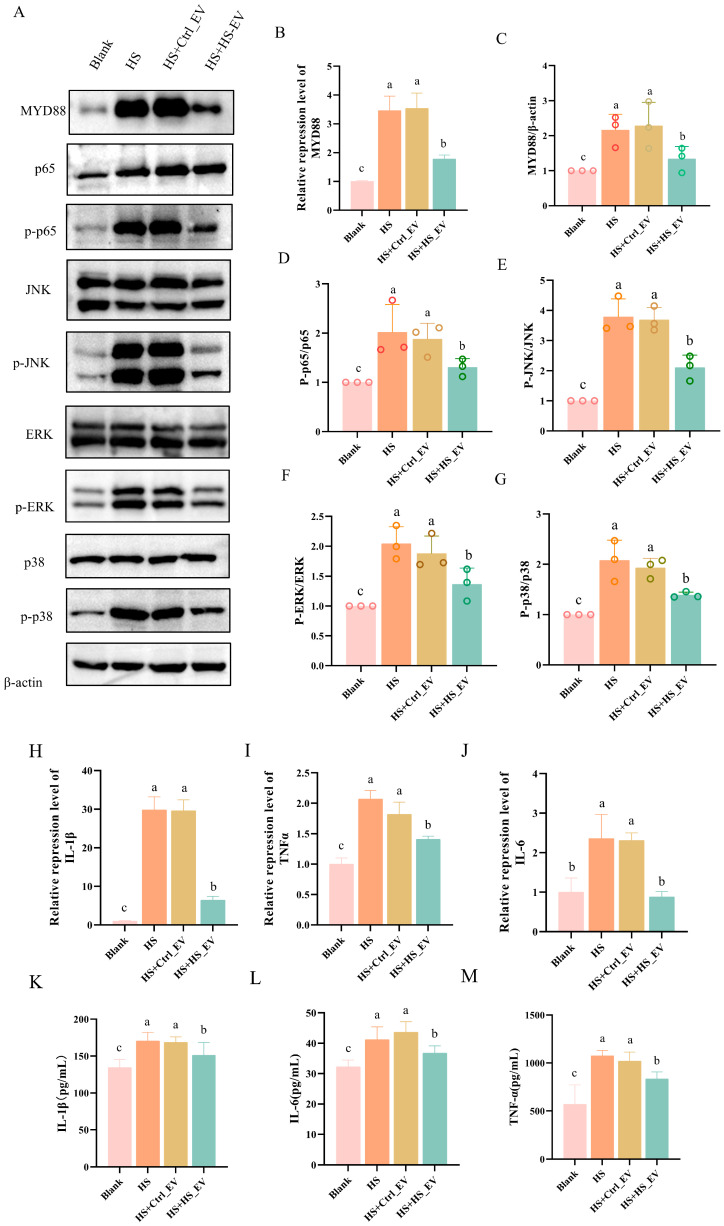
HS_EV attenuates heat stress-induced inflammatory signaling and cytokine production in primary hepatocytes. (**A**) Western blot analysis of MYD88, p65, p-p65, JNK, p-JNK, ERK, p-ERK, p38, p-p38, and β-actin (*n* = 3). (**B**,**C**) Relative MYD88 mRNA and protein expression levels. (**D**–**G**) Quantification of the p-p65/p65, p-JNK/JNK, p-ERK/ERK, and p-p38/p38 ratios. Western blot analyses were performed using three independent primary hepatocyte experiments (*n* = 3). (**H**–**J**) Relative mRNA expression levels of IL-1β, IL-6, and TNF-α (*n* = 4). (**K**–**M**) Concentrations of IL-1β, IL-6, and TNF-α in the culture supernatant (*n* = 8). Data are presented as mean ± SD. Different lowercase letters indicate significant differences among groups (*p* < 0.05).

Based on the transcriptomic results indicating suppression of TLR-related signaling, the expression of the adaptor molecule MYD88, the activation status of its downstream NF-κB and MAPK pathways, and the production of pro-inflammatory cytokines were further examined in primary hepatocytes. Heat stress significantly increased both the mRNA and protein expression levels of MYD88 compared with the Blank group. A similar pattern was observed in the HS + Ctrl_EV group, whereas HS + HS_EV treatment significantly reduced MYD88 expression relative to both the HS and HS + Ctrl_EV groups (Figure 9A–C).

Consistent with the changes in MYD88 expression, heat stress markedly increased the phosphorylation levels of p65, ERK, JNK, and p38, as reflected by significantly elevated p-p65/p65, p-ERK/ERK, p-JNK/JNK, and p-p38/p38 ratios compared with the Blank group, indicating activation of both the NF-κB and MAPK signaling pathways. Similar activation patterns were observed in the HS + Ctrl_EV group. In contrast, HS + HS_EV treatment significantly decreased the phosphorylation levels of p65, ERK, JNK, and p38 relative to the HS and HS + Ctrl_EV groups, although these levels remained higher than those in the Blank group (Figure 9D–G).

To further assess the downstream inflammatory response, the expression and secretion of the pro-inflammatory cytokines IL-1β, IL-6, and TNF-α were measured. qRT-PCR analysis showed that heat stress significantly increased the mRNA levels of all three cytokines compared with the Blank group. This increase was not obviously attenuated in the HS + Ctrl_EV group, whereas HS + HS_EV treatment significantly reduced the transcript levels of IL-1β, IL-6, and TNF-α (Figure 9H–J). Similarly, ELISA analysis of the culture supernatant demonstrated that heat stress significantly increased the secretion of these cytokines, while HS + HS_EV treatment significantly decreased their release compared with both the HS and HS + Ctrl_EV groups (Figure 9K–M).

### 3.11. MYD88 Overexpression Weakens the Protective Effects of HS_EV in Heat-Stressed Primary Hepatocytes

**Figure 10:**
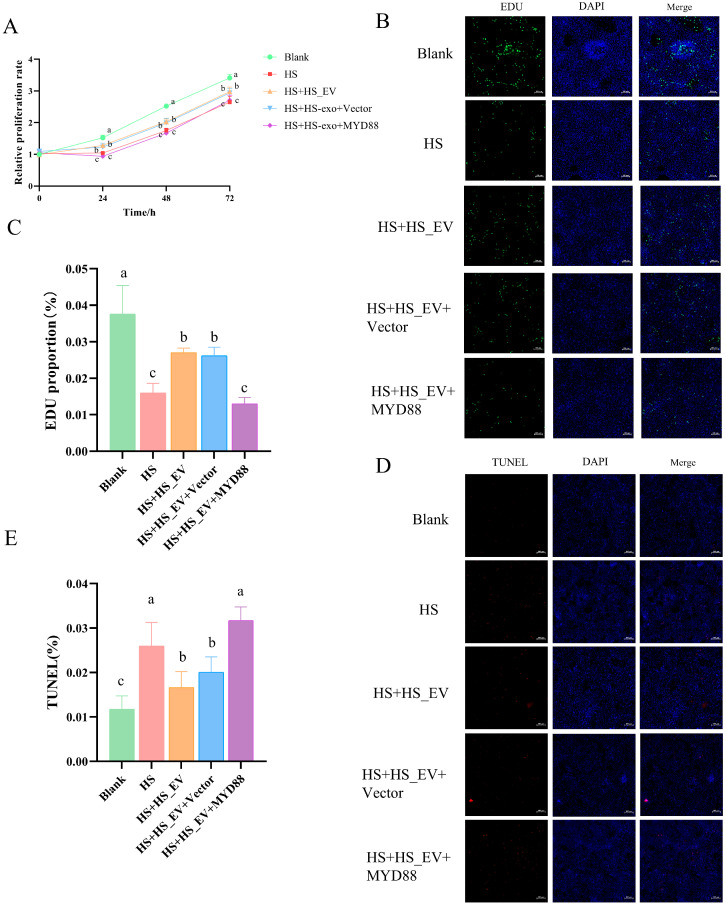
MYD88 overexpression weakens the protective effects of HS_EV on heat-stressed primary hepatocytes. (**A**) Relative proliferation rate (*n* = 8). (**B**) Representative EdU fluorescence images (*n* = 3). (**C**) Quantification of EdU-positive cells (*n* = 3). (**D**) Representative TUNEL fluorescence images (*n* = 3). (**E**) Quantification of TUNEL-positive cells (*n* = 3). Data are presented as mean ± SD. Different lowercase letters indicate significant differences among groups (*p* < 0.05). Scale bar = 100 μm.

To further determine whether suppression of MYD88 is required for the protective effects of HS_EV, primary hepatocytes were divided into five groups: Blank, HS, HS + HS_EV, HS + HS_EV + Vector, and HS + HS_EV + MYD88. CCK-8 analysis showed that heat stress markedly inhibited hepatocyte proliferation compared with the Blank group. HS_EV treatment partially restored proliferative capacity, and the Vector group showed a similar trend. In contrast, MYD88 overexpression weakened the protective effect of HS_EV, as reflected by a reduced proliferation rate compared with the HS + HS_EV and HS + HS_EV + Vector groups (Figure 10A).

Consistent with the CCK-8 results, EdU staining showed that heat stress significantly decreased the proportion of proliferating hepatocytes relative to the Blank group. This reduction was partially alleviated by HS_EV treatment, whereas the Vector control showed no obvious additional effect. However, MYD88 overexpression markedly reduced the proportion of EdU-positive cells compared with the HS + HS_EV and HS + HS_EV + Vector groups, indicating that restoration of MYD88 impaired the pro-proliferative effect of HS_EV (Figure 10B,C).

Similarly, TUNEL staining demonstrated that heat stress significantly increased hepatocyte apoptosis. HS_EV treatment reduced the proportion of TUNEL-positive cells, whereas the Vector group remained similar to the HS + HS_EV group. In contrast, MYD88 overexpression increased the proportion of apoptotic cells relative to the HS + HS_EV and HS + HS_EV + Vector groups, indicating that the anti-apoptotic effect of HS_EV was markedly attenuated after MYD88 restoration (Figure 10D,E).

### 3.12. MYD88 Overexpression Weakens the Inhibitory Effects of HS_EV on Inflammatory Signaling in Heat-Stressed Primary Hepatocytes

**Figure 11:**
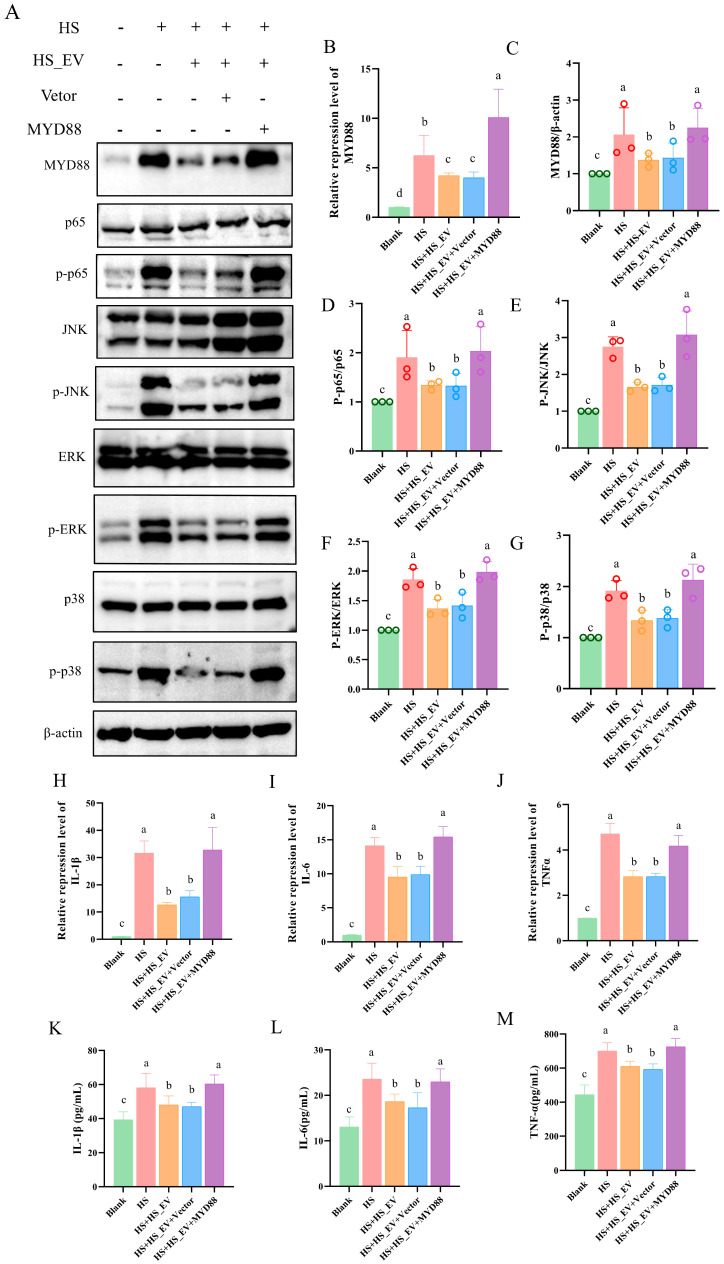
MYD88 overexpression weakens the inhibitory effects of HS_EV on inflammatory signaling and cytokine production in heat-stressed primary hepatocytes. (**A**) Western blot analysis of MYD88, p65, p-p65, JNK, p-JNK, ERK, p-ERK, p38, p-p38, and β-actin (*n* = 3). (**B**,**C**) Relative MYD88 mRNA and protein expression levels. (**D**–**G**) Quantification of the p-p65/p65, p-JNK/JNK, p-ERK/ERK, and p-p38/p38 ratios. (**H**–**J**) Relative mRNA expression levels of IL-1β, IL-6, and TNF-α (*n* = 4). (**K**–**M**) Concentrations of IL-1β, IL-6, and TNF-α in the culture supernatant (*n* = 8). Data are presented as mean ± SD. Different lowercase letters indicate significant differences among groups (*p* < 0.05).

Compared with the Blank group, heat stress markedly increased both the mRNA and protein expression levels of MYD88. This increase was attenuated by HS_EV treatment, whereas the Vector group showed a pattern similar to that of the HS + HS_EV group. In contrast, MYD88 overexpression increased MYD88 expression again, indicating that restoration of MYD88 counteracted the suppressive effect of HS_EV on this adaptor molecule (Figure 11A–C).

Consistent with the changes in MYD88 expression, heat stress markedly enhanced the phosphorylation levels of p65, ERK, JNK, and p38, as reflected by increased p-p65/p65, p-ERK/ERK, p-JNK/JNK, and p-p38/p38 ratios relative to the Blank group. These changes were reduced in the HS + HS_EV group and remained similar in the HS + HS_EV + Vector group. However, MYD88 overexpression elevated the phosphorylation levels of these signaling molecules again, indicating reactivation of the NF-κB/MAPK signaling pathways (Figure 11D–G).

To further examine the downstream inflammatory response, the expression and secretion of IL-1β, IL-6, and TNF-α were evaluated. Heat stress significantly increased the mRNA levels of all three cytokines, and this increase was markedly attenuated by HS_EV treatment. A similar trend was observed in the HS + HS_EV + Vector group. In contrast, MYD88 overexpression increased the transcript levels of IL-1β, IL-6, and TNF-α again (Figure 11H–J). Likewise, ELISA analysis showed that the elevated secretion of these cytokines induced by heat stress was reduced by HS_EV, but this inhibitory effect was weakened after MYD88 overexpression (Figure 11K–M).

### 3.13. PMA Treatment Weakens the Protective Effects of HS_EV on Heat-Stressed Primary Hepatocytes

**Figure 12:**
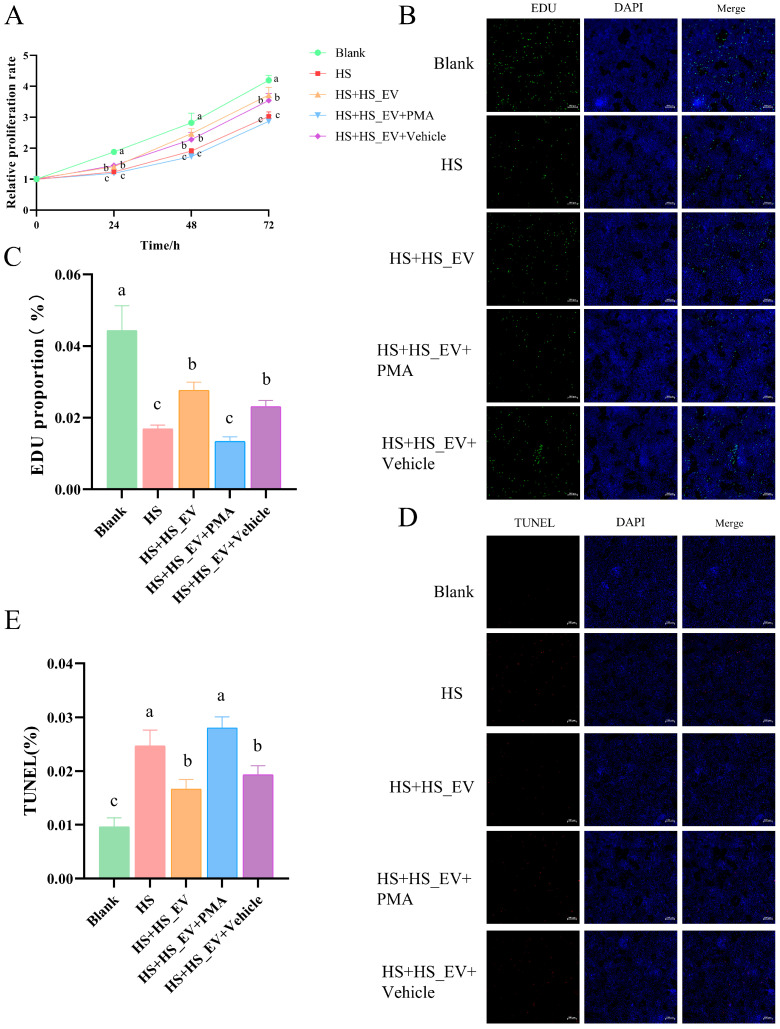
PMA treatment weakens the protective effects of HS_EV on heat-stressed primary hepatocytes. (**A**) Relative proliferation rate (*n* = 8). (**B**) Representative EdU fluorescence images (*n* = 3). (**C**) Quantification of EdU-positive cells (*n* = 3). (**D**) Representative TUNEL fluorescence images (*n* = 3). (**E**) Quantification of TUNEL-positive cells (*n* = 3). PMA, NF-κB activator. Data are presented as mean ± SD. Different lowercase letters indicate significant differences among groups (*p* < 0.05). Scale bar = 100 μm.

To further determine whether reactivation of inflammatory signaling could counteract the protective effects of HS_EV, primary hepatocytes were divided into five groups—Blank, HS, HS + HS_EV, HS + HS_EV + Vehicle, and HS + HS_EV + PMA—with PMA used as an NF-κB activator. CCK-8 analysis showed that heat stress markedly inhibited hepatocyte proliferation compared with the Blank group. HS_EV treatment partially restored proliferative capacity, whereas the Vehicle group showed a similar trend. In contrast, PMA treatment weakened the beneficial effect of HS_EV, as reflected by a lower proliferation rate than that observed in the HS + HS_EV group (Figure 12A).

Consistent with the CCK-8 results, EdU staining showed that heat stress significantly reduced the proportion of proliferating hepatocytes relative to the Blank group. This reduction was partially alleviated by HS_EV treatment, and the Vehicle control showed no obvious additional effect. However, PMA treatment decreased the proportion of EdU-positive cells compared with the HS + HS_EV and HS + HS_EV + Vehicle groups, indicating that pathway reactivation impaired the pro-proliferative effect of HS_EV (Figure 12B,C).

Similarly, TUNEL staining demonstrated that heat stress significantly increased hepatocyte apoptosis. HS_EV treatment reduced the proportion of TUNEL-positive cells, whereas the Vehicle group remained broadly comparable to the HS + HS_EV group. In contrast, PMA treatment increased the proportion of apoptotic cells relative to the HS + HS_EV and HS + HS_EV + Vehicle groups, indicating that the anti-apoptotic effect of HS_EV was weakened after pharmacological reactivation of inflammatory signaling (Figure 12D,E).

### 3.14. PMA Treatment Partially Reverses the Inhibitory Effects of HS_EV on Inflammatory Signaling and Cytokine Production in Heat-Stressed Primary Hepatocytes

**Figure 13:**
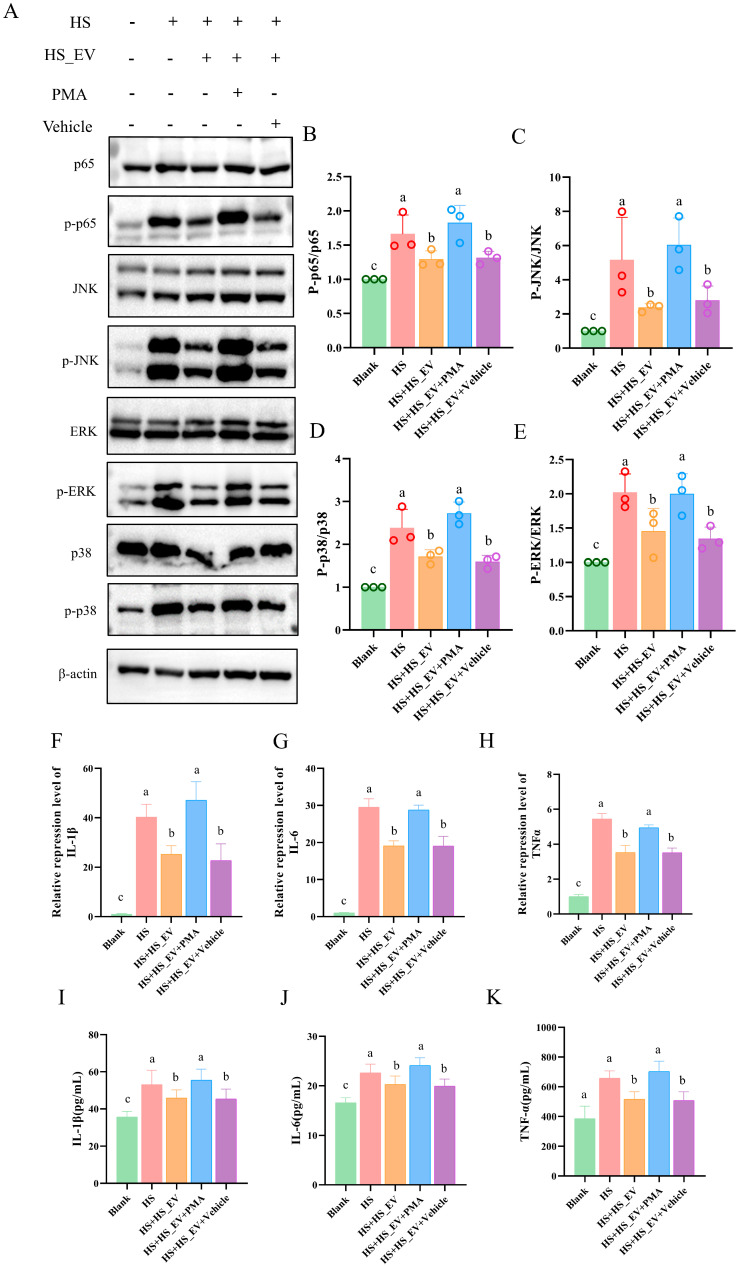
PMA treatment partially reverses the inhibitory effects of HS_EV on inflammatory signaling and cytokine production in heat-stressed primary hepatocytes. (**A**) Western blot analysis of p65, p-p65, JNK, p-JNK, ERK, p-ERK, p38, p-p38, and β-actin (*n* = 3). (**B**–**E**) Quantification of the p-p65/p65, p-JNK/JNK, p-p38/p38, and p-ERK/ERK ratios (*n* = 3). (**F**–**H**) Relative mRNA expression levels of IL-1β, IL-6, and TNF-α (*n* = 4). (**I**–**K**) Concentrations of IL-1β, IL-6, and TNF-α in the culture supernatant (*n* = 8). Data are presented as mean ± SD. Different lowercase letters indicate significant differences among groups (*p* < 0.05).

Heat stress significantly increased the phosphorylation levels of p65, ERK, JNK, and p38, as reflected by elevated p-p65/p65, p-ERK/ERK, p-JNK/JNK, and p-p38/p38 ratios relative to the Blank group. These changes were attenuated in the HS + HS_EV group and remained largely comparable in the HS + HS_EV + Vehicle group. However, PMA treatment increased the phosphorylation levels of these signaling molecules again, indicating reactivation of the NF-κB/MAPK signaling pathways (Figure 13A–E).

To further examine the downstream inflammatory response, the expression and secretion of IL-1β, IL-6, and TNF-α were evaluated. qRT-PCR analysis showed that heat stress significantly increased the mRNA levels of all three cytokines, whereas HS_EV treatment reduced their expression. The Vehicle group showed no obvious additional effect. In contrast, PMA treatment increased the transcript levels of IL-1β, IL-6, and TNF-α again (Figure 13F–H). Similarly, ELISA analysis demonstrated that the heat stress-induced secretion of these cytokines was attenuated by HS_EV, but this inhibitory effect was weakened after PMA treatment (Figure 13I–K).

## 4. Discussion

Heat stress is widely recognized as a systemic challenge that disrupts physiological homeostasis and compromises organ function [1,35,36]. In the present study, heat-stressed chickens exhibited elevated rectal temperature, marked alterations in biochemical, metabolic, and inflammatory indices, indicating that thermal exposure induced a pronounced systemic stress response accompanied by metabolic disturbance and inflammatory activation. These findings are consistent with previous reports showing that heat stress in chickens disrupts immune homeostasis, endocrine and metabolic balance, and inflammatory status at the whole-body level [35,37,38]. Histopathological examination further demonstrated that heat stress caused mild-to-moderate lesions in multiple organs, including the heart, spleen, lung, kidney, and particularly the liver, which displayed hepatic steatosis, cytoplasmic vacuolization, inflammatory foci, and venous congestion. This observation agrees with recent studies describing heat stress as a multi-organ pathological process driven by oxidative stress, inflammatory injury, and metabolic dysfunction, with the liver being especially vulnerable because of its central roles in metabolism and immunoregulation [36]. Collectively, these findings support the view that heat stress induces systemic injury in chickens and further identify the liver as a major target organ during thermal challenge.

Against this background of systemic stress, circulating EVs may represent an important component of the host response to heat exposure. In this study, plasma-derived EVs from control and heat-stressed chickens displayed characteristic vesicular morphology, expected size distribution, and EV-associated marker expression, consistent with current EV characterization criteria. Notably, heat stress significantly increased total EV protein content in plasma, suggesting enhanced EV release under stress conditions. This result agrees with growing evidence that temperature- and other stress-related stimuli can alter EV production, cargo composition, and biological activity [39,40]. Moreover, circulating EVs preferentially accumulated in the liver in vivo and were efficiently internalized by primary hepatocytes in vitro. These findings are consistent with previous studies showing that systemically administered extracellular vesicles are frequently enriched in the liver and can mediate functional cargo delivery to hepatocytes, supporting their role as biologically active carriers rather than passive by-products of stress [41,42,43]. Taken together, these data suggest that plasma EVs may participate in systemic adaptation to heat stress by delivering stress-associated signals to hepatocytes, with the liver representing a major target organ of EV-mediated intercellular communication during thermal challenge [42].

A major finding of the present study was that heat stress-derived plasma EVs exerted a clear protective effect on primary hepatocytes exposed to heat stress. HS_EV significantly restored proliferative capacity and reduced apoptosis in heat-stressed hepatocytes, whereas Ctrl_EV showed little protective effect. These observations support the emerging concept that stress-conditioned extracellular vesicles can function as adaptive signals that enhance recipient-cell stress tolerance, rather than merely reflecting donor-cell injury. Similar phenomena have been reported in other systems, in which stress-derived EVs attenuated apoptosis, oxidative stress, or mitochondrial dysfunction in recipient cells [44,45]. In addition, recent studies indicate that extracellular vesicles can promote hepatocyte proliferation and liver repair in a context-dependent manner [46]. Therefore, HS_EV may represent an adaptive circulating signal generated during heat stress that helps hepatocytes better tolerate subsequent thermal injury.

The omics data further suggest that HS_EV-associated hepatoprotection is closely associated with extensive molecular reprogramming in recipient hepatocytes. Transcriptomic analysis showed that HS_EV activated glutathione metabolism and other metabolic pathways, while suppressing apoptosis-, p53-, cytokine-, Toll-like receptor-, and NOD-like receptor-related signaling. This pattern is consistent with previous studies indicating that EVs regulate recipient liver cells through coordinated effects on inflammation, apoptosis, and metabolism, rather than through a single downstream axis [47,48]. Our non-coding RNA profiling and enrichment analyses further supported this interpretation by repeatedly highlighting pathways related to inflammation, stress responses, apoptosis, and metabolism. Thus, HS_EV appears to reshape the hepatocyte response program toward reduced inflammatory injury and improved metabolic adaptation, in line with the broader view that EV cargo can function as an integrated regulator of hepatic stress responses [49].

Among the pathways implicated by these analyses, the MYD88/NF-κB/MAPK axis emerged as the most directly validated mechanism in the present study. Heat stress significantly increased both the mRNA and protein expression of MYD88 and promoted phosphorylation of p65, JNK, ERK, and p38, indicating activation of both NF-κB and MAPK signaling. HS_EV markedly attenuated all of these changes, whereas Ctrl_EV did not exert a comparable inhibitory effect. These findings agree with previous studies showing that heat stress activates MYD88-dependent inflammatory signaling in poultry tissues and promotes downstream NF-κB and MAPK responses associated with oxidative injury, cytokine production, and apoptosis [50,51]. Because MYD88 is a key adaptor linking stress- and danger-related signals to downstream inflammatory cascades, our data support the interpretation that HS_EV alleviates hepatocyte injury, at least in part, by dampening MYD88-dependent amplification of inflammatory signaling. This view is also consistent with growing evidence that EVs can suppress inflammatory signaling through cargo-dependent regulation of NF-κB-related pathways [52,53]. Importantly, the rescue experiments further strengthened this mechanistic interpretation. MYD88 overexpression weakened the protective effects of HS_EV on hepatocyte proliferation and apoptosis, and also attenuated its inhibitory effects on MYD88/NF-κB/MAPK activation and inflammatory cytokine production. Likewise, pharmacological reactivation of NF-κB-related signaling by PMA partially reversed the beneficial effects of HS_EV on proliferation, apoptosis, and inflammatory output. Together, these findings provide additional functional evidence that suppression of MYD88/NF-κB/MAPK-associated inflammatory signaling is not merely correlated with, but contributes to, HS_EV-mediated hepatoprotection.

Consistent with this interpretation, HS_EV also significantly reduced the expression and secretion of the major pro-inflammatory cytokines IL-1β, IL-6, and TNF-α in heat-stressed hepatocytes. These downstream changes provide functional evidence that attenuation of MYD88/NF-κB/MAPK signaling was biologically meaningful and translated into reduced inflammatory output. This result is consistent with previous studies showing that heat stress enhances pro-inflammatory cytokine production in poultry tissues through TLR/MYD88-related signaling and thereby aggravates tissue injury [51]. Moreover, recent EV studies have shown that EV treatment can reduce inflammatory cytokine output and ameliorate liver injury in different pathological contexts, including decreases in TNF-α and IL-1β after intervention [49,54,55]. Taken together, our results support a model in which HS_EV attenuates heat stress-induced hepatocyte injury by modulating inflammatory signaling at multiple levels, including upstream adaptor expression, downstream kinase activation, and terminal cytokine production.

Despite these promising findings, several limitations should be acknowledged. First, histopathological evaluation was performed using tissues from only four chickens per group, which may limit the statistical robustness of the morphological assessment, although these observations were generally consistent with the physiological, biochemical, and inflammatory alterations observed in the same model. Second, although our in vivo experiments demonstrated the preferential hepatic distribution of circulating HS_EV, the functional and mechanistic validation in this study was mainly conducted in primary hepatocytes in vitro. Therefore, the in vivo hepatoprotective effects of exogenous HS_EV under heat stress conditions remain to be further established. Third, although MYD88/NF-κB/MAPK-associated inflammatory signaling was identified as an important pathway involved in HS_EV-mediated protection, the specific EV cargo molecules responsible for these effects remain to be determined. Given the complexity of the transcriptomic, non-coding RNA, and ceRNA results, the hepatoprotective effect of HS_EV is likely to depend on the coordinated action of multiple EV components rather than on a single molecule. Future studies combining cargo profiling with gain- and loss-of-function approaches will help to clarify the molecular basis of EV-mediated adaptation to heat stress.

## 5. Conclusions

In conclusion, the present study demonstrates that heat stress promotes the release of plasma-derived extracellular vesicles (EVs) in chickens and that these EVs preferentially accumulate in the liver and are readily internalized by primary hepatocytes. More importantly, HS_EV alleviates heat stress-induced hepatocyte injury by restoring proliferative capacity, reducing apoptosis, and attenuating inflammatory signaling, which is associated with reduced activation of the MYD88/NF-κB/MAPK axis. These protective effects are accompanied by broad transcriptomic and non-coding RNA reprogramming, indicating that circulating EVs participate in adaptive intercellular communication during thermal stress. Collectively, our findings identify HS_EV as a potential endogenous mediator of hepatoprotection under heat stress conditions and provide a basis for future studies aimed at developing EV-based strategies to mitigate heat stress-induced injury in poultry.

## Figures and Tables

**Table 1:** Clean reads of rRNA-depleted libraries from primary hepatocytes.

Sample	Raw_Reads	Raw_Bases	Clean_Reads	Clean_Bases	Q20	Q30	Unique_Map
Ctrl_EV1	91,407,018	13.71 G	88,746,486	13.31 G	99.28	96.86	85.57%
Ctrl_EV2	90,986,856	13.65 G	88,573,456	13.29 G	99.29	96.87	85.06%
Ctrl_EV3	95,768,492	14.37 G	93,928,818	14.09 G	99.01	96.09	81.84%
Ctrl_EV4	88,529,264	13.28 G	86,793,220	13.02 G	99.07	96.19	83.60%
Ctrl_EV5	94,119,774	14.12 G	92,084,814	13.81 G	99.21	96.67	83.67%
HS_EV1	93,330,916	14.00 G	91,520,018	13.73 G	99.20	96.59	83.70%
HS_EV2	92,331,294	13.85 G	90,989,154	13.65 G	99.05	96.14	83.89%
HS_EV3	94,207,754	14.13 G	92,643,966	13.90 G	99.03	96.16	83.71%
HS_EV4	95,463,514	14.32 G	93,702,084	14.06 G	99.23	96.67	83.61%
HS_EV5	102,816,888	15.42 G	100,645,186	15.10 G	99.23	96.63	83.54%

**Table 2:** Clean reads of small RNA libraries from primary hepatocytes.

Sample	Reads	Clean_Reads	Q20	Q30	GC Content	Mapped sRNA
Ctrl_EV1	11,734,710	11,138,259	99.19	97.62	55.02	7,228,440 (93.96%)
Ctrl_EV2	11,438,843	10,812,336	99.49	98.29	54.83	7,204,102 (93.47%)
Ctrl_EV3	11,941,609	11,242,936	99.44	98.06	55.85	6,887,975 (94.02%)
Ctrl_EV4	11,723,750	11,148,922	99.44	97.98	56.06	7,249,774 (94.17%)
Ctrl_EV5	11,880,702	11,391,346	99.5	98.30	55.46	7,700,609 (93.32%)
HS_EV1	11,391,566	10,999,202	99.55	98.32	53.10	9,125,031 (93.73%)
HS_EV2	11,666,949	11,164,907	99.48	98.12	53.28	8,213,938 (94.19%)
HS_EV3	11,519,448	11,028,037	99.56	98.41	53.03	7,073,319 (96.22%)
HS_EV4	11,994,516	11,485,596	99.52	98.37	53.50	7,588,739 (95.88%)
HS_EV5	11,482,733	10,946,349	99.50	98.28	53.58	8,094,359 (93.55%)

## Data Availability

The datasets presented in this study are available in the NCBI Sequence Read Archive (SRA) under BioProject accession numbers PRJNA1443036 and PRJNA1447393.

## Abbreviations

The following abbreviations are used in this manuscript:

HS	Heat stress
miRNA	MicroRNA
lncRNA	Long non-coding RNA
circRNA	Circular RNA
ceRNA	Competing endogenous RNA
CDS	Coding sequence
UTR	Untranslated region
GO	Gene Ontology
KEGG	Kyoto Encyclopedia of Genes and Genomes
GSEA	Gene Set Enrichment Analysis
NF-κB	Nuclear factor kappa-B
MAPK	Mitogen-activated protein kinase
ERK	Extracellular signal-regulated kinase
JNK	c-Jun N-terminal kinase
p38	p38 mitogen-activated protein kinase
TLR	Toll-like receptor
MYD88	Myeloid differentiation primary response 88
NOD	Nucleotide-binding oligomerization domain
p53	Tumor protein p53
CCK-8	Cell Counting Kit-8
EdU	5-ethynyl-2′-deoxyuridine
qPCR	Quantitative real-time PCR
WB	Western blot
NTA	Nanoparticle tracking analysis
TEM	Transmission electron microscopy

## Appendix A

**Table A1:** miRNA reverse transcription and qPCR primer sequences.

Primer Name	Primer Sequence (5′-3′)
gga-miR-30c-5p-RT	GTCGTATCCAGTGCAGGGTCCGAGGTATTCGCACTGGATACGACAGCTGA
gga-miR-30c-5p-F	GCGCGTGTAAACATCCTACACTC
gga-miR-30c-5p-R	AGTGCAGGGTCCGAGGTATT
gga-miR-1456-5p-RT	GTCGTATCCAGTGCAGGGTCCGAGGTATTCGCACTGGATACGACGCGCGG
gga-miR-1456-5p-F	AAAGGACGGAGGCGGC
gga-miR-1456-5p-R	AGTGCAGGGTCCGAGGTATT
gga-miR-203a-RT	GTCGTATCCAGTGCAGGGTCCGAGGTATTCGCACTGGATACGACCAAGTG
gga-miR-203a-F	CGCGGTGAAATGTTTAGGAC
gga-miR-203a-R	AGTGCAGGGTCCGAGGTATT
gga-miR-425-3p-RT	GTCGTATCCAGTGCAGGGTCCGAGGTATTCGCACTGGATACGACGACAGA
gga-miR-425-3p-F	CGCATCGGGGATGTCGTG
gga-miR-425-3p-R	AGTGCAGGGTCCGAGGTATT
gga-miR-184-3p-RT	GTCGTATCCAGTGCAGGGTCCGAGGTATTCGCACTGGATACGACACCCTT
gga-miR-184-3p-F	CGCGTGGACGGAGAACTGAT
gga-miR-184-3p-R	AGTGCAGGGTCCGAGGTATT
gga-miR-146a-5p-RT	GTCGTATCCAGTGCAGGGTCCGAGGTATTCGCACTGGATACGACAACCCA
gga-miR-146a-5p-F	CGCGTGAGAACTGAATTCCA
gga-miR-146a-5p-R	AGTGCAGGGTCCGAGGTATT
5S-F	CCATACCACCCTGGAAACGC
5S-R	TACTAACCGAGCCCGACCCT

**Table A2:** Primers used for qPCR.

Primer Name	Primer Sequence (5′-3′)
MX1-F	GCCTCTGCCATTAGGCTGC
MX1-R	CATGCTGCTGCCTCATCCT
MYD88-F	CCTCGGCCTTTACCTCAACC
MYD88-R	CTGTTCCATGCCCCACGCT
USP2-F	CTGTTCCATGCCCCACGCT
USP2-R	ACCACCTTGGAGCTCTTGGTC
TXNRD1-F	GTGCGAAGTAACCGCAGAGA
TXNRD1-R	GTTCCTCCAAGACCCCATGAA
FTH1-F	TTCCTGCGTCAACAGTGCTT
FTH1-R	CCGGTCAAAATAGTAGGACATGC
PHGDH-F	TCGATGTCTTCACACAGGAGC
PHGDH-R	TGGCCATGTCCACTATCTGC
>TCONS_42007-F	TCCAGTGTCTCACCCTTCTCA
>TCONS_42007-R	TAGATGCAAGCTAGGCTGTCC
>TCONS_102258-F	AGATTGCCTTCAGCCTGCAT
>TCONS_102258-R	GCGCTTGCTTTGTGTTGGTA
>TCONS_81984-F	AAGCAGCTCGGCAAATTTGG
>TCONS_81984-R	CAAAGAGGAGGAGCTGTGCA
>TCONS_92784-F	CAGCTGCTCAGTGAGTCCAT
>TCONS_92784-R	ATTCTGTGGCTGGACGTCTG
>TCONS_41058-F	ACCCCAGACCTGACCCTATC
>TCONS_41058-R	GTTCAGTGGCCCAGTCTTGT
>TCONS_6663-F	TTGTCCTGCAGTCCTGTTCC
>TCONS_6663-R	GGGAAAAGACCTCGTCTGCA
>novel_circ_0002760-F	ACTCGCTGTCCTCTGGAACT
>novel_circ_0002760-R	GGCAACATCAGGTCGTTTCC
>novel_circ_0008005-F	TCAGAACGAGAGATGGGGGA
>novel_circ_0008005-R	TCAGTCCCAGCACAGTTCAC
>novel_circ_0002596-F	AAAGGGAGAGGATTGAAGGCC
>novel_circ_0002596-R	TCATCCTTGGGGTCATCCTG
>novel_circ_0000209-F	ATGGAGACGTGGTGATTCCA
>novel_circ_0000209-R	GCTCGGCAGCTTAAAGACAC
>novel_circ_0014635-F	GCACGGCTAGCAAAGGAAAA
>novel_circ_0014635-R	TGGCCAAACTTCTGGGATGT
>novel_circ_0011894-F	GGTATGGAGCAAAAGGAAGCG
>novel_circ_0011894-R	TTTGCTTTTGGTTTGTTTTCACCC
β-actin-F	CAGCCAGCCATGGATGATGA
β-actin-R	CATACCAACCATCACACCCTGA
